# Learning better together? A scoping review of in-person interprofessional undergraduate simulation

**DOI:** 10.1186/s41077-025-00351-5

**Published:** 2025-04-29

**Authors:** Brona Joyce, Davina Carr, Alison Smart, Dakota Armour, Gerard J. Gormley

**Affiliations:** 1 Centre for Biomedical Sciences Education, School of Medicine, Dentistry and Biomedical Sciences, Queen’s University Belfast, Belfast, Northern Ireland; 2 Centre for Medical Education, School of Medicine, Dentistry and Biomedical Sciences, Queen’s University Belfast, Belfast, Northern Ireland; 3 School of Nursing and Midwifery, Queen’s University Belfast, Belfast, Northern Ireland

**Keywords:** Simulation, Interprofessional, Undergraduate, Education

## Abstract

**Background:**

Given the increasing complexity of contemporary clinical practice, there has never been a more important time to provide interprofessional educational (IPE) activities across the learning continuum to develop collaborative practice. From the outset of health professional training, it is crucial that students not only develop their own professional skills but also gain an awareness of the capabilities of other healthcare professionals and how best to work collaboratively. Despite simulation being a common teaching modality in many undergraduate curricula, little is known about the range of interprofessional activities within these settings. Therefore, this study aims to address the following research question: What is known about undergraduate in-person (IP) simulation-based education (SBE)?

**Methods:**

We conducted a scoping literature review, adhering to the PRISMA-ScR extension guidelines, and used the Arksey and O’Malley framework. Our search covered three electronic databases: Web of Science (WOS), MEDLINE, and Embase. We utilised Covidence systematic review software to assist in screening articles. To support data charting, we developed a data extraction tool and employed both qualitative and quantitative techniques through numerical and thematic analysis to ensure a comprehensive representation of our data.

**Results:**

A total of 97 studies were included, with most publications originating from the USA, UK, and Australia. Two main themes emerged regarding the impact of IP SBE at an individual level: confidence and role identification. Several themes related to the impact on teams included knowledge of other professional roles/values, communication, and teamwork. The studies identified various barriers and enablers to simulation, particularly logistical barriers and financial challenges associated with complex technologically enabled simulation. Faculty collaboration and resources were reported as primary enablers in facilitating the delivery of simulation activities.

**Conclusions:**

The impact of IP-SBE on learners and interprofessional teams is predominantly positive, with reported benefits including increased confidence, enhanced role identification, and improved communication and teamwork skills. However, challenges such as logistical barriers and resource constraints highlight the need for collaborative faculty efforts and adequate infrastructure to support IP-SBE implementation. Despite the growing interest in IP-SBE, there remains a notable lack of standardised reporting on simulation design and debriefing processes in both teaching practice and research.

**Supplementary Information:**

The online version contains supplementary material available at 10.1186/s41077-025-00351-5.

## Background

Educators face increasing challenges in effectively preparing healthcare professionals (HCPs) and students for the realities of contemporary clinical practice. This preparation involves not only addressing the advancing clinical and technical complexities of modern healthcare but also navigating the social complexities of working within dynamic and evolving multidisciplinary teams. Beyond individual contributions, HCPs must collaborate effectively to provide the highest quality care for patients. In recognition of this need, HCP regulators and other organisations are increasingly mandating interprofessional education (IPE) in their curricula, beginning at an undergraduate level [1, 2]. With such drivers, there is an increasing need to provide and enhance IPE opportunities for students and healthcare professionals. However, enabling such interprofessional learning is not without its challenges and is considered by many to be a ‘wicked problem’ (a sociocultural issue that is difficult to solve due to its complex and interconnected nature) in health professions education (HPE) [3]. In order to attempt to ‘tame’ this wicked problem, IPE opportunities need to be expanded and most importantly at an undergraduate level, when HCP students begin to develop their professional identities and collaborative abilities.

### Interprofessional education

Mirroring the collaborative nature of real clinical practice, learning should also have a collaborative focus. In its broadest sense, IPE has been defined as ‘occasions when members or students of two or more professions learn with, from, and about each other, to improve collaboration, and the quality of care and services’ [1]. Evidence would indicate that IPE can have a positive impact on the provision of healthcare by improving interactions between different professionals, enhancing patient safety, and streamlining treatment processes for patients [4]. When individuals from different professional backgrounds come together to work as a team, different learning attitudes, styles, and opinions can come together synergistically [5, 6]. Moreover, it is important to note the beneficial impact IPE has on patient outcomes [7]. When HCPs are familiar with IPE learning, it can lead to enhanced communication between members of the HCP team, having a positive impact on patient care [8]. IPE in health HPE can take many forms, including classroom-based learning, online environments, clinical workplace experiences, and simulation settings. Importantly, laying the foundation for collaborative practice should begin at the undergraduate level, as healthcare professionals start to embed their professional skills and identities.

### Simulation-based education

Simulation is a form of experiential learning and is popular in HPE [9]. In this form of teaching, learners gain new insights and knowledge from facilitated experiences and guided conversations which aim to transform their competencies [10]. Often focusing at an individual level, there is increasing interest in how SBE can enhance team-based competencies across the learning continuum, including undergraduate, postgraduate, and continual professional development. SBE can take many forms, including virtual-based modalities (such as AR/VR) and in-person simulation (e.g. manikin or simulated participant-based simulations). Given the resource-intensive nature of SBE, organisations need to make informed decisions about when to best utilise SBE and the different forms of SBE. This is of particular importance for in-person simulation, given the complexities of organising individuals to be present at specific times and ensuring they have a meaningful learning opportunity [11, 12]. Moreover, when we introduce more than one profession in an SBE activity — additional complexities are introduced that must be navigated. In an undergraduate setting, managing large cohorts of learners, there are often competing demands between different curricular structures and requirements, making it challenging to bring HCP students together in one place, at one time, for shared learning experiences.

Despite the growth of simulation in the undergraduate sector, and the recognised importance of nurturing collaborative practice at this stage of learning, the evidence base remains sparse on how interprofessional simulation-based education (IP-SBE) is implemented in undergraduate settings. Furthermore, while studies have explored in-person IP — in undergraduate contexts, a systematic overview is needed to understand the nature, extent, and impact of this form of learning [13]. Therefore, in this study, we aimed to address the following research question: What is known about undergraduate in-person interprofessional SBE? Specifically, we wanted to explore the format and nature of such undergraduate-based IP-SBE activities, the impact on learners, and any insights into the organisational enablers/barriers to delivering this complex form of learning.

## Methods

### Methodological approach

Considering the nature of our research aim, we chose to adopt a scoping literature review methodology. This approach allowed us to map the terrain of the evidence base regarding in-person IP-SBE in the undergraduate arena. Furthermore, it enabled us to identify important knowledge gaps that will guide future lines of research inquiry. We followed the PRISMA-ScR extension for our scoping review (Appendix 1) [14].

### Research team and reflexivity

In keeping with the IPE context of this study and our constructivist stance in this study, we ensured diversity within our research team. B. J. is an undergraduate human biology student, D. C. is a general practitioner (GP) and clinical teaching fellow, A. S. is a lecturer in nursing specialising in simulation, D. A. is a specialist foundation programme doctor, and G. G. is a GP and simulation scholar. Throughout the study, the team met regularly, engaging in reflexive discussions and note-taking to maintain team reflexivity and alignment with the study’s aims.

### Scoping literature review method

We followed the Arksey and O’Malley methodological framework to guide our scoping review, namely: Stage 1: Defining the review question; Stages 2 and 3: Developing the search strategy and selection criteria; Stage 4: Charting the data; and Stage 5: Collating, summarising, and reporting the results. Due to time and resource constraints, we pragmatically decided not to undertake Stage 6 (the consultation exercise) of this framework [15].

### Stage 1: Defining the research question

To define our research question, we applied the ‘Population, Situation’ tool [16]: namely the *population* was undergraduate HCP students, and the *situation* was in-person IP-SBE. Specifically, our objectives in this study were to establish the following [16]:

1. What is known about the features of in-person IP-SBE in the HCP undergraduate arena?
2. The impact, if any, of such in-person IP-SBE activities
3. Factors that enable, or inhibit, such a form of learning in undergraduate healthcare profession curricula?

### Stages 2 and 3: Search strategy and selection criteria

Underpinned by our research objectives, we devised a search strategy with the assistance of a subject-specific librarian, as members of the research team collaborated to construct search terms that were refined through preliminary searches and further consultation with the librarian (see Tables 1 and 2).

**Table 1:** Search terms used for MEDLINE and Embase database searches

Search terms used for MEDLINE and Embase	
**Set**	**Term**
1	simulation*.mp
2	sim.mp
3	simulation based education.mp
4	SBE.mp
5	undergraduate*.mp
6	Interprofessional Education/
7	IPE.mp
8	healthcare education.mp
9	HCE.mp
10	interprofessional*.mp
11	1 or 2 or 3 or 4
12	Undergraduate student.mp
13	5 or 12
14	6 or 7 or 8 or 9 or 10
15	11 and 13 and 14

**Table 2:** Search terms used for Web of Science database search

Search terms used for world of science	
Set	Term
	Simulation* or sbe or sim (All fields)
And	undergraduate* (All fields)
And	interprofessional* or ipe or HCE or “healthcare education (All fields)

In total, three electronic databases were selected for our search: Web of Science (WOS), MEDLINE, and Embase. These databases were chosen given their relevance to healthcare and healthcare education. The search was conducted in November 2023. The search terms used in each database can be seen in Tables 1 and 2.

Searches from the three online databases were imported into Covidence Systematic Review Software^©^ (Melbourne, Australia, 2024) to assist in article screening. A total of *726* papers were initially imported: 259 from WOS, 239 from MEDLINE, and 228 from Embase. After identifying and removing 292 duplicates, *434* studies were screened based on title and abstract. This screening was carried out by two members of the team (B. J. and D. C.), and any conflicts that arose were settled by a third member of the team (G. G.) and finally agreed upon by consensus. In keeping with the iterative nature of scoping review methodology, a priori inclusion and exclusion criteria were devised and refined throughout the selection process. The following criteria were applied in our selection process:

### Inclusion criteria

1. Educational activity that pertained to healthcare
2. Educational activity was simulation based.
3. Learners at an undergraduate level
4. Involved at least two healthcare professions

### Exclusion criteria

1. To keep our findings relevant and contemporaneous, we excluded papers published before January 2014 (i.e. within the last 10 years).
2. Focusing on in-person simulation, we excluded virtual methods of simulation (e.g. online simulation, VR/AR simulation, gaming technology).
3. Papers that were conference abstracts, commentary pieces, review protocols, conference reports, short notes, e-posters, technical reports, oral abstracts, narrative pieces, or letters, given the likelihood of these not providing sufficient evidence to address our research objectives

After title and abstract screening was completed, *170* papers moved into a full-text review. Following the application of our exclusion criteria, *97* papers were deemed in scope to be assessed in our literature review.

### Stage 4: Charting the data

The research team developed a data charting extraction tool using a Microsoft Excel® spreadsheet (Supplement file 1). The tool’s categories were aligned with the research objectives, specifically study details and characteristics, the nature and characteristics of the IP-SBE activity, the impact of the in-person IP-SBE activity, and organisational factors (including enablers and inhibitors) in implementing the in-person IP-SBE activity. B. J., D. A., and G. G. conducted a calibration exercise and refinement of the extraction tool to ensure consistency in data extraction (i.e. regular meetings with open discussion and testing of the extraction tool until the group agreed consensus). Once consensus was reached, B. J. and D. A. extracted the data, with periodic checks and verification by the other researchers. Any disagreements were resolved through consensus among the entire research team.

### Stage 5: Collating, summarising, and reporting the results

Given the variety of our data, we employed both qualitative and quantitative techniques to synthesise and present our findings. We conducted numerical analysis to examine the characteristics of the reviewed articles and in-person IP-SBE activities. For qualitative data analysis, we adopted a thematic approach to present our findings, drawing insights from categorised data. This method required collaborative and iterative efforts to ensure a comprehensive representation of our data.

## Results

### Selection of evidence sources

**Fig. 1:**
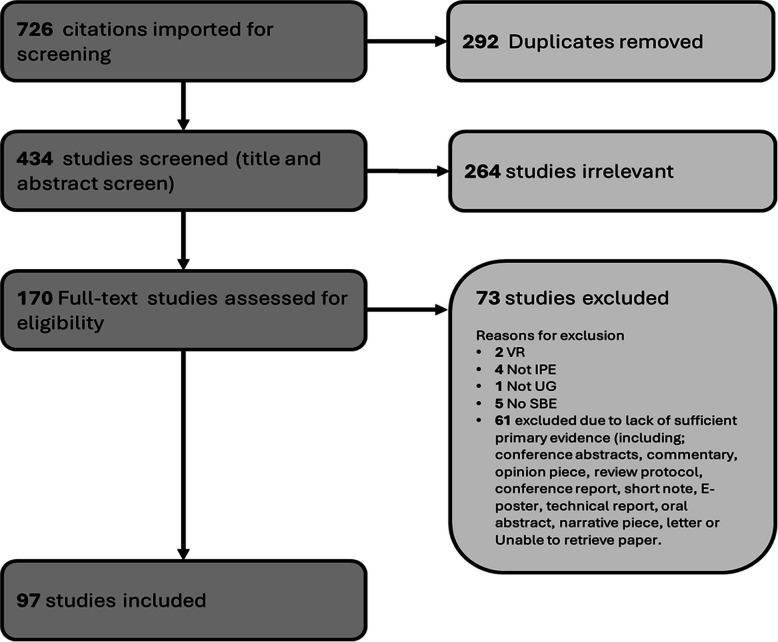
PRIMSA flowchart of publications retried, screened, and extracted

The PRIMSA flowchart (Fig. 1) illustrates the number of articles that were retrieved, screened, and extracted in our study. See supplementary file 1 for a list of articles in this scoping review.

### Characteristics of extracted evidence sources

Table 3 summarises the main characteristics of the publications included in our study in terms of their country of origin and study methodology.

**Table 3:** Main characteristics of evidence sources (*n* = 97)

	**Number (*n*)**	**Percentage (%)**
**Country where study was conducted**		
USA	38	39.2
UK	16	16.5
Australia	10	10.3
South Africa	5	5.2
Germany	4	4.1
Canada	4	4.1
Sweden	4	4.1
Switzerland	2	2.1
China	2	2.1
Turkey	2	2.1
India	2	2.1
South Korea	2	2.1
Other	6	6.2
**Study methodology**		
Mixed methodology	27	27.8
Qualitative study	17	17.5
Quantitative study	9	9.3
Survey	8	8.2
Systematic review	6	6.2
Descriptive study	5	5.2
Scoping review	5	5.2
Other^a^	20	20.6

^a^Other studies included reviews (scoping, systematic, literature) and survey style studies

**Fig. 2:**
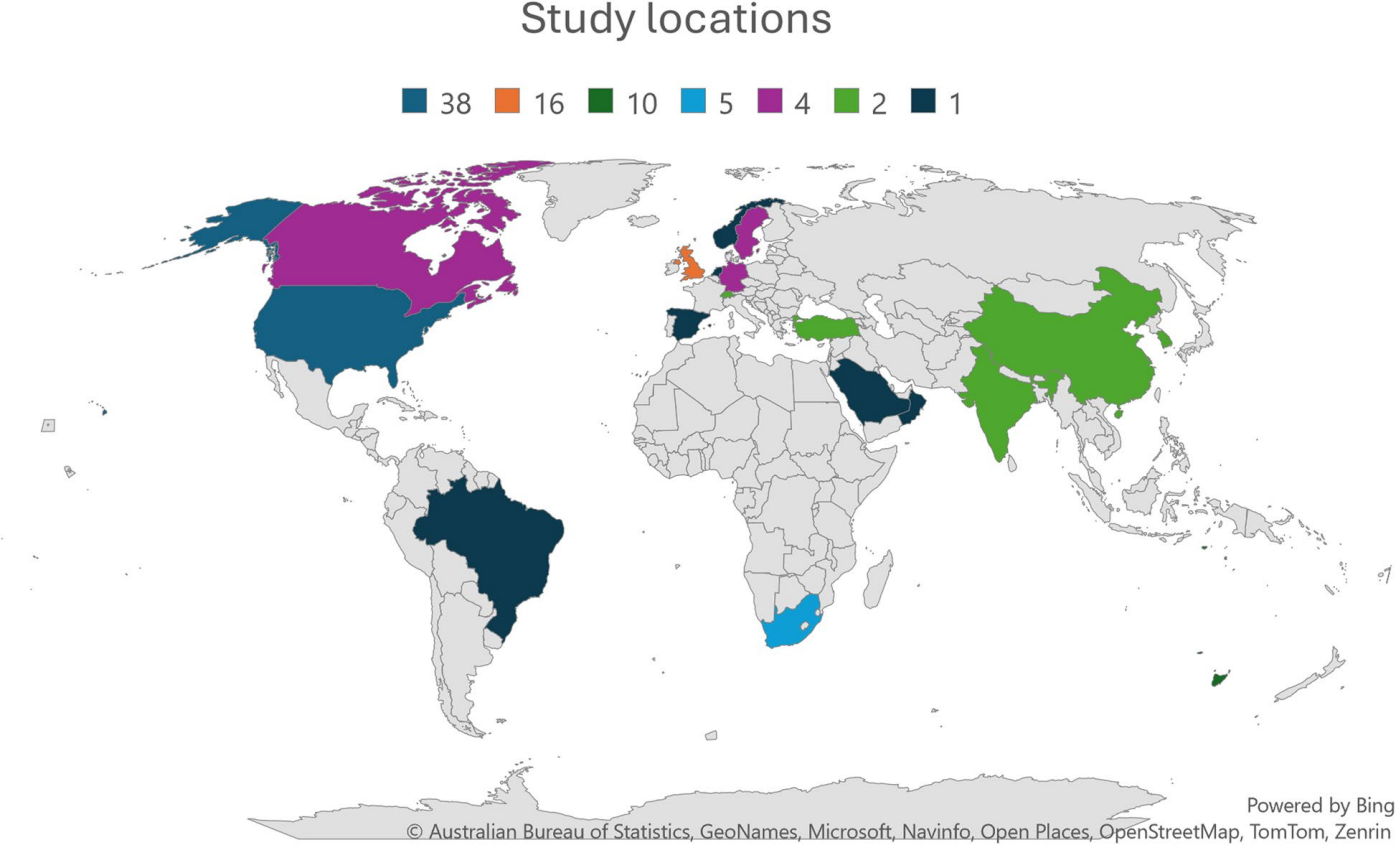
Pictograph of study locations in publications included in scoping review (Absolute number of countries not a % of total: USA 38; UK 16; Australia 10; South Africa 5; Germany, Canada, and Sweden 4;Switzerland, China, Turkey, India, and South Korea 2; the Netherlands, Brazil, Saudi Arabia, Spain, Norway, and Oman 1)

The majority of publications originated from a few countries, specifically the USA (39.2%, 38/97), the UK (16.5%, 16/97), and Australia (10.3%, 10/97) (refer to Fig. 2 for a pictograph of study locations.)

The majority of studies selected (71.1%, 69/97) were published since 2018. Mixed methods (27.8%, 27/97) were the most common methodology used, followed by systematic reviews (6.2%, 6/97) and scoping reviews (5.2%, 5/97).

### Characteristics of IP-SBE activities described in evidence sources

A wide range of undergraduate in-person IP-SBE activities were described in the publications selected for our study. Table 4 summarises the main features of these IP-SBE activities.

**Table 4:** Main features of the IP-SBE activities mentioned in evidence sources (*n* = 97)

Participating professions in the IP-SBE activity	Number (*n*)	Percentage (%)
Medicine + nursing	30	31.0
Medicine + nursing + pharmacy	4	4.1
Medicine + midwifery	3	3.1
Nursing + midwifery	2	2.1
Medicine + pharmacy	2	2.1
Nursing + pharmacy	2	2.1
Nursing + respiratory therapy	2	2.1
Other^a^	52	53.6
**Simulation modality used in the IP-SBE activity**	**Number (*n*)**	**Percentage (%)**
Full-body manikin-based simulation	27	27.8
Simulated participant-based simulation	24	24.7
Task manikin-based simulation	4	4.1
Mixture	16	16.5
Other methods^b^	6	6.2
N/A^c^	20	20.6
**Simulation activity design**	**Number (*n*)**	**Percentage (%)**
Co-designed between professions	54	55.7
Uni-professional design	5	5.2
No mention	15	15.5
N/A^d^	23	23.7
**Format of debrief in IP SBE activity**	**Number (*n*)**	**Percentage (%)**
Interprofessional debrief	20	20.6
No mention of nature of debriefers	41	42.3
No mention of debriefing	20	20.6
N/A^e^	16	16.5

^a^There was a wide spread of ‘combinations of professions’; the following is listed as ‘other’ as no combination appeared more than once: (dietician + psychology + nursing + occupational therapy), (dieticians + optometry + OT + medicine + nursing + physiotherapy), (medicine + nursing + clinical medical officer + ophthalmic clinical officer), (medicine + nursing + paramedics), (medicine + nursing + pharmacy + operating department practitioner), (medicine + nursing + physician assistant + respiratory therapy + clinical laboratory science + physical therapy + nuclear medicine technology), (medicine + nursing + physiotherapy), (medicine + nursing + physiotherapy + social work), (medicine + nursing + radiography), (medicine + nursing + social worker), (medicine + anaesthesia technician), (medicine + dentistry + pharmacy + health + rehabilitation science), (medicine + nursing + nutrition dietician + social work), (medicine + nursing + pharmacy + physiotherapy + occupational therapy + dentistry), (medicine + nursing + pharmacy + physiotherapy + social work), (medicine + nursing + social work (PG) + chaplaincy (PG)), (medicine + operating room technician), (medicine + police), (medicine + nursing + physiotherapy+ exercise physiology + allied health assistance + occupational therapy + podiatry), (midwifery + anaesthesia (PG)), (midwifery + paramedic), (nursing + athletic training + homeland security studies), (nursing + clinical laboratory science), (nursing + dental hygienist), (nursing + dentistry + pharmacy), (nursing + paramedics + radiography + surgical technology), (nursing + radiological science + physicians associate), (nursing + respiratory therapy), (nursing + social work), (nursing + social work + physical therapy + doctor of nursing), (nursing + speech and language), (nutrition + dietetics + occupational therapy + physiotherapy + optometry + nursing + medicine), (paramedics + occupational therapy (physiotherapy + nursing), (public health + informatics + biomedical sciences + medicine + nursing) (public health education majors + health studies majors), (respiratory therapy + medicine + nursing)

^b^Other’ simulation included papers in which the mode of simulation was not documented

^c^N/A’: Studies where simulation activity design was not specifically discussed, including systematic reviews, scoping reviews, and literature reviews

^d^N/A’: Studies where simulation activity design was not specifically discussed, including systematic reviews, scoping reviews, and literature reviews

^e^N/A’ included systematic reviews, scoping reviews, and literature reviews, where debrief as a topic may have been discussed but was not directly from a simulation activity

The number of professions in the IP-SBE activity varied from 2 to 7 [17, 18]. The IP-SBE activities described in the studies were predominantly composed of medicine and nursing professionals (31%, 30/97). A wide range of other professional combinations were also described, primarily involving medicine, nursing, pharmacy, physiotherapy, and social work. Notably, additional professions such as Clinical Laboratory Science and Nuclear Medicine Technology also participated in the IP-SBE activities cited in the evidence. A few studies included interprofessional interactions between undergraduate students and postgraduate professionals for the purpose of IP-SBE. For example, Peterson et al. included first-year medical residents in their IP-SBE activities [19].

Simulations using a full-body manikin (FBM) were the most common teaching modality used in the evidence sources selected in this scoping review (27.8%, 27/97). Studies that utilised simulated participants (SPs) closely followed (24.7%, 24/97). In other evidence sources, a mixture of simulation modalities was used in 16.5% (16/97) of studies, combining SPs and FBMs. The remaining 6.2% (6/97) were categorised as ‘other’, including studies where the mode of simulation was not specifically documented.

Most notably, learning objectives for the IP-SBE were poorly recorded in many of the studies (33%, 32/97), with a significant portion not stating any learning objectives at all. Among those that did document intended learning objectives, a majority (49.4%, 48/97) focused on communication skills or clinical skills. Specifically, 20.6% (20/97) cited interprofessional practice (IP) as a learning outcome.

### Co-designed features of simulation

The majority of simulation activities described in the studies were co-designed between professions (55.7%, 54/97), while only 5.2% (5/97) of the studies were designed uni-professionally. Design features of simulation were not mentioned in 15.5% (15/97) of the papers.

An aspect of simulation that was highlighted for its importance in a number of papers was debriefing [20]; however, only 63% (61/97) of the studies detailed the nature of the debrief or commented on some aspect of briefing after the IP-SBE activity. Only 21% (20/97) of the studies explicitly mentioned that the debriefing session was co-facilitated. Video recordings from the simulation activity were utilised in a number of studies (22%, 21/97) to aid in the debriefing process. These recordings allowed all participants and facilitators to comment and enabled students to reflect on their own actions shown in the video [21, 22]. Studies with detailed information about the use of debriefing mentioned that the debriefers had received specific training beforehand [23]. One study highlighted the importance of both co-facilitated debriefing and individual profession-based debriefing. The co-facilitated debriefing allowed for discussions on interprofessional communication and team-based skills, while the individual profession-based debriefing facilitated discussions on specific clinical skills related to each profession [19]. A wide range of debriefing models were used [24, 25]

### Impact of the IP-SBE activity

In terms of the impact of the IP-SBE activities on learners, two main themes were derived: namely confidence [25–32] and role identification [19, 33–39]. One study particularly noted students’ weaknesses in a pretest, with role clarity identified as the main theme. Following the IP-SBE, a posttest indicated improvement in role clarity for two-thirds of the students [40]. Numerous themes came through about the impact the IP-SBE had on the interprofessional team aspect of the SBE activity — including knowledge of the other professions’ role and value in healthcare [18, 21, 22, 25, 27, 31–35, 37, 38, 41–62], and communication and teamwork [17, 36, 44, 51, 53, 56, 58, 60, 62–70]. Interestingly, Uslu-Sahan reported that none of the students in the study had participated in any form of IP-SBE before, and only 66.7% had participated in SBE previously. The results showed that all participants exhibited increased perception and attitude of teamwork after completing the IP-SBE [71]. In another study, the authors reported negative impacts of poor communication skills among interprofessional teams during the IP-SBE activity [72]. The study noted that students lacked knowledge of formal communication tools, such as the use of SBAR (situation, background, assessment, recommendation) [72].

### Barriers and enablers

Many of the challenges identified in the studies centred around logistical barriers. Coordinating timetables to enable students from different professions to participate in the same simulation activity, at the same location, and at the same time was frequently cited as a recurring issue. Another reported challenge was the nature of student participation in IP-SBE: some professions were required to complete the simulation activity as a mandatory component of their studies, while others had the option to participate voluntarily [40, 68]. Many studies reported the use of complex technologically enabled forms of simulation (e.g. complex FBM), together with some mentioning the financial challenges associated with it. Grants and specific funding sources were identified as enablers in some studies for providing IP-SBE activities. A pilot study using simulation was curtailed due to financial constraints associated with implementing the IP-SBE [44]. The majority of studies reported faculty collaboration and resources as the main enablers in facilitating the delivery of simulation activities. One study highlighted both these factors and voluntary participation as key to the successful implementation of IP-SBE activities [68].

## Discussion

This scoping review maps the contours of the evidence base regarding in-person undergraduate simulation involving IP HCP students. The findings of this study provide an overview of an emerging educational tool for the future of healthcare by identifying study trends, characterising simulation activities, exploring co-designed features of simulation, and assessing the impact of simulation activities. Additionally, information surrounding the organisational features in delivering IP-SBE was mapped, allowing insights in how best to implement such educational opportunities.

### Study trends

Like the nature of simulation research, it was no surprise that the majority of studies consisted of mixed-method approaches, allowing for comprehensive exploration of various aspects of IP-SBE. Data collection was predominantly quantitative, often using pre- and posttests including Likert scales. The top three countries emerging with studies related to IP-SBE were the USA, the UK, and Australia, indicating the established presence of IP-SBE in these regions. In contrast, a study published in Turkey, one of two studies included from this country in the scope, identified the lack of IP-SBE initiatives, highlighting the importance of future inclusion [71].

### The ins and outs of the simulation activities

There was a vast array of combinations of professions within IP-SBE. The predominant combination was medicine and nursing. However, the combination of medicine and nursing does not accurately reflect current healthcare practice. The inclusion of more specialised healthcare professions such as Clinical Laboratory Science and Nuclear Medicine Technology demonstrates the versatility that IP-SBE can have. This suggests that with adaptations, simulation activities can be tailored to include any profession as required [19, 26]. Simulation scenarios involving emergency medical situations were commonly recorded, which either signifies one of two things: this area of healthcare urgently requires educational methods like IP-SBE, or the nature of simulation-based scenarios naturally lends itself to an emergency setting [25, 73]. Other scenarios included poverty, falls, cultural appreciation, palliative care, and ethical dilemmas, demonstrating the adaptability of IP-SBE to various contexts to meet learners’ needs. The main specialties frequently reported in simulation activities were emergency medicine, obstetrics and gynaecology, care of the elderly, palliative care, and surgery.

Given the significant number of studies that did not report intended learning objectives of their simulation activities, it is crucial to thoroughly explain these activities in research. While some researchers may prioritise other aspects of IP-SBE and consider reporting learning objectives unnecessary, this omission creates a notable gap in the literature when synthesising studies.

It is noteworthy that over half of the papers indicated that simulation activities were co-designed among multiple professions. When developing IP-SBE, it is crucial for all involved professions to participate in the simulation creation process to ensure that the needs of each profession are identified and met [25].

An aspect of IP-SBE that is gaining much focus is co-debriefing; however, as stated earlier, many studies failed to report the debriefing process or provide detailed information on its delivery. Some studies provided thorough explanations of how and by whom the debriefing was conducted, noting that facilitators often received formal training in debriefing techniques. No comparative studies were found that evaluated trained facilitators versus untrained ones for debriefing. Various methods of debriefing were identified, including those utilising video technology, peer-led debriefs, and formal debriefing protocols/tools such as ‘MAES standards’, ‘Delta Plus framework’, and ‘Steinwachs’ [45]. The majority of studies did not mention any specific model of debriefing. Among those studies that did not report debriefing, it remained unclear whether debriefing actually occurred or simply not documented in the paper. This raises the question of whether the importance of debriefing is underappreciated by some researchers in this field.

Another consistent issue observed regarding debriefing in simulations was the lack of documentation about who facilitated the debriefing. This raises concerns about the integrity of the IP-SBE experience. If the goal is to learn collaboratively across professions, why would the debriefing be facilitated by only one person? Without appropriate representation in the debriefing process, certain professions may miss out, affecting their learning experience [74].

Overall, the studies reviewed suggest that the literature lacks common standards and protocols for facilitating debriefing post IP-SBE. This gap highlights an area for future research.

### Impact of undergraduate IP-SBE

We reviewed and identified the impact of simulation activities on students both as individuals and as part of an interprofessional (IP) team. The results were predominantly positive. Across the literature, self-efficacy was frequently recognised, with students increasing their self-awareness of abilities and limitations in their own knowledge. This was primarily assessed through pre- and posttests administered before and after the simulation, respectively. Additionally, students self-reported several themes in free-text responses following the simulation activities, including increased confidence and clearer role identification.

When examining the impact of simulation activities on students as part of an IP team, we identified major themes related to knowledge of other professions’ roles and values, as well as communication and teamwork. Increased understanding of the roles and values of peers within the team fostered collaboration and enhanced team efficacy, both of which contribute to improved patient care. Furthermore, improved IP collaboration led to enhanced communication skills among students in a supportive learning environment [62].

While the majority of recorded impacts were positive, it is important to note some negative experiences and potential drawbacks. One negative impact reported was that junior midwives expressed discomfort participating in role-play activities. This reluctance was more prevalent among junior midwives compared to their senior counterparts, suggesting that experienced students may be more willing to engage in simulation activities. However, the discomfort felt by junior midwives could potentially deter them from participating in IP-SBE if they are forced to engage in activities that make them feel uncomfortable [75]. Careful consideration in the design of the simulation activity is vital to ensure students feel comfortable and therefore achieve the best possible from the learning experience [76]. While the literature frequently discusses the impact of role identification, a design issue in crafting simulation activities was noted. Some scenarios involved assigning students to pre-engineered roles to ensure structured teamwork and facilitate smoother simulations. However, this approach does not mirror real-life situations where team dynamics often evolve naturally, with leaders emerging organically.

If the goal of IP-SBE is to replicate real-life scenarios, could assigning roles hinder students from assuming their natural positions within an IP team? [36].

### The organisational features that allow IP-SBE to happen

Recurring themes contributing to the success of IP-SBE included interprofessional faculty collaboration, the availability of simulation resources, and the creation of psychologically safe learning environments. Many logistical barriers must be addressed first, such as arranging for students to be released from placements or classes to participate in activities. Due to the nature of IP-SBE, participation often occurs in small groups with limited resources, which can complicate the facilitation of IP-SBE for all students.

Collaboration between different faculties to plan and design simulation activities, involving students from each profession, allowed this to happen. This collaboration also ensured that all participants had an active role in the simulation, meeting specific learning objectives within their professional domains. Students repeatedly commented on the IP-SBE using high-fidelity simulations as very realistic and improving their learning experience compared to simulations using lower-technical resources [65]. The mode of participation was frequently highlighted; students engaging in the same simulation activity may have varying attitudes towards the session, with some attending voluntarily and others being mandated to participate. Many simulation activities, such as high-fidelity simulations, can be resource-intensive and require expensive equipment. This limits access for students in developed or economically disadvantaged areas.

### Limitations

Despite the novelty and methodologically rigorous approach of this literature review, the findings must be considered within the study’s limitations. Given the nature of scoping reviews, it is challenging to ensure the inclusion of all relevant literature on the topic. Although we were guided by a subject-specific librarian and conducted methodical searches across three databases, which yielded a substantial number of papers for a comprehensive review, it is possible that some relevant studies were not included.

We intentionally excluded papers focused on postgraduate or qualified individuals to focus on IP-SBE at the undergraduate level, aiming to understand how it educates the next generation of HCPs. Research on how IP-SBE develops qualified HCP would be worthy of future research. Additionally, we limited our review to papers published within the last 10 years to maintain current evidence, though this approach might have excluded relevant earlier publications.

Challenges were encountered in identifying the characteristics of IP in such forms of SBE. Moving forward, further research is essential to establish criteria for documenting features of IP-SBE activities, including learning objectives and outcomes, design characteristics, nature of facilitation, pre-briefing, and debriefing processes, to enable clear reporting of successful practices. While medicine and nursing were prominently featured, it is crucial to expand IP-SBE activities across other professions to ensure that students are prepared for the evolving healthcare landscape.

Finally, Stage 6, the ‘consultation exercise’, of the scoping review methodology by Arksey and O’Malley was not conducted due to time and resource constraints. Additionally, exploratory research involving simulation-based educators would be valuable for further exploring IP-SBE in an undergraduate setting.

## Conclusion

In conclusion, this scoping review highlights the growing recognition of the importance of collaborative learning among undergraduate HCPs and the necessity of preparing them for multidisciplinary teamwork in clinical practice. Through a detailed examination of the literature, this review identifies key trends, characteristics of undergraduate IP-SBE activities, and their impacts on learners and interprofessional teams. Co-designed simulations and interprofessional debriefing sessions emerge as critical elements for fostering effective learning experiences.

The impact of IP-SBE on learners and interprofessional teams is predominantly positive, with reported benefits including increased confidence, enhanced role identification, and improved communication and teamwork skills. However, challenges such as logistical barriers and resource constraints underscore the need for collaborative faculty efforts and adequate infrastructure to support IP-SBE implementation. Despite the growing interest in IP-SBE, there remains a notable lack of standardised reporting on simulation design and debriefing processes.

This review’s findings have significant implications for healthcare education practice, emphasising the need for standardised reporting guidelines and standards of simulation practice across healthcare facilities to ensure consistency and quality in IP-SBE implementation. Lastly, identified gaps in the literature suggest several avenues for future research, including exploring the impact of IP-SBE on postgraduate healthcare professionals and addressing challenges related to resource allocation and logistical coordination.

## Supplementary Information

Additional file 1. Supplement file 1: List of articles in the scoping review.

Additional file 2. Appendix 1: Preferred Reporting Items for Systematic reviews and Meta-Analyses extension for Scoping Reviews (PRISMA-ScR) Checklist.

## Data Availability

No datasets were generated or analysed during the current study.
