# Supplementary material for: Learning better together? A scoping review of in-person interprofessional undergraduate simulation
Source: Adv Simul (Lond). 2025 Apr 29;10:24. doi: 10.1186/s41077-025-00351-5 (PMC12042576; doi:10.1186/s41077-025-00351-5)
Supplement: Supplementary file 1 — Additional file 1. Supplement file 1: List of articles in the scoping review. [file 41077_2025_351_MOESM1_ESM.pdf]

Study Details									Simulation Activity				Impact of IP-SBE			Organisational Factors			Comments	Systamatic review/ Sccoping review infromation
Study number	Author	Title	Journal	Year	Study location	Aims of study	study type	Professions	Modality of simulation	Learning objective of simulation	Context of the simulation activity	Nature of debrief (IPE)	Impact of IPE simulation on individual	Impact of simulation on collaborative practice / working	Other impact	Challenges in delivering IPE sim	Enablers in delivering IPE Sim	Co-designed (IPE) Sim or one profession joining in on another's		
1	Huth	Establishing trust within interprofessional teams with a novel simulation activity in the pediatric clerkship	Journal of interprofessional care	2020	US	To develop, implement, and evaluate a simulation activity intended to promote interprofessional trust and conflict resolution in the context of a pediatric clerkship rotation	Prospective Review	Medicine + Nursing	Simulated participant	1. To apply the VALUED mnemonic to facilitate conflict resolution and trust building. 2. To recognize the importance of shared decision making and common goals in patient care. 3. To reflect on one's own role and contribution to the interprofessional team.	interprofessional conflict between the student and the nurse about a patient care decision	facilitated by the nurse, another nurse educator, and two pediatricians	medical students were reported to have improved confidence to constructively (88%) and respectfully (76%) manage disagreements.	Students were reported to have enhanced communication (89%) and conflict resolution (89%) among memebrs of the IPE team. 92% of medical students reported having endorsed the effectiveness of nurses role.	It was reported that the sim activity achieved the learning objectives by 86%.	n/a	Partnership with the Boston Children's Hospital Simulator Program, the medical school, the pediatrics clerkship, and the nursing faculty = collaborated in curriculum development, scenario design, and facilitation of the simulation and debriefing sessions.	Co-designed		
2	Becker	Changes in medical students' and anesthesia technician trainees' attitudes towards interprofessionality - experience from an interprofessional simulation-based course	BMC medical education	2022	Munich	To investigate the effects of an interprofessional simulation course on final year medical students and final year anesthesia technician trainees' attitudes towards interprofessional collaboration and learning, and to understand the reasons for these effects.	Mixed methodology	Medicine + Anesthesia technician	Mixture		Simulations of critical incidents or emergency cases in the operating room or emergency department.	IP teams of physician + 1 nurse or anesthsia technician	Self-assessment of communication and teamwork skills improved in both professions.	Attitudes towards interprofessional learning improved only in medical students, not in ATT. Attitudes towards interprofessional interaction and interprofessional relationships improved in both	Qualitative data revealed teamwork, communication , hierarchy and the perception of one's own and other health profession as main topics that might underlie the changes in participants' attitudes.	IP diferncces in attitudes towards IPL. Lack of cummunicationbetween different HP conflicting perceptions of team work. Mixed view on hieracrhy in medicine	Not noted	Co-designed		
3	Baker	Undergraduate cardiac arrest team training	The Clinical Teacher	2015	UK	The aim of this study is to evaluate the impact of interdisciplinary team-based (ITB) versus individual discipline-based (DB) learning with multiple health care disciplines during a simulated cardiac arrest.	Randomised control trial	Nursing + Radiological science + Physicians associate	Task manikin	Practice collaboration while completing key resuscitative tasks prior to the arrival of medical emergency response teams (5 Mins sim).	A simulated patient experiencing a cardiac arrest before and after being educated using the First 5 Minutes Curriculum.	During debriefing, participants were allowed to write subjective comments regarding their experience, and these were grouped thematically by author. No detail about who facilitated debrief or of what nature.	Students in the ITB sim showed greater improvement in their knowledge of caridac arrset care that those inn DB sim.	70% students said they enjoyed wokring with other disciplines and suggested more in their learning .	students in the ITB sim suggested follow up sessions with IDT of each disicipline	Coordinating schedules between faculty members and students of several disciplines.	1. The First-5 Curriculum. 2. The Patient Simulation Center. 3. The Single Educator.	Not Noted.		
4	Son	The Effects of Simulation Problem-Based Learning on the Empathy, Attitudes toward Caring for the Elderly, and Team Efficacy of Undergraduate Health Profession Students.	International journal of environmental research and public health	2021	Switzerland	The aim of this study is to investigate the effect of an undergraduate course that applied simulation problem-based learning (S-PBL) on nursing and dental hygiene students' empathy, attitudes toward caring for the elderly, and team efficacy	Experimental study	Nursing + Dental hygienest	Full body manikin	S-PBL aimed to enhance students' empathy, attitudes toward caring for the elderly, and team efficacy through interactions and collaboration between nursing and dental hygiene students	S-PBL involved small group activities using an elderly care manikin in a simulated clinical environment, where students performed basic care and identified medical errors that posed a threat to patient safety.	50 min debrief proceeded with the instructor's feedback and the students' self-evaluations and reflections to conclude the entire program.	Empathy did not change significantly after participating in the simulation problem-based learning (S-PBL) compared to before participation. Attitudes toward caring for the elderly improved significantly after participating in the S-PBL compared to before participation.	Team efficacy improved significantly after participating in the S-PBL compared to before participation.		covid-19 limited students time to particapte in IPE sim	use of small gorup SPBL, manikinn	Designed by a professor in the school of nursing		
5	James	Nursing student's attitudes toward teams in an undergraduate interprofessional mass casualty simulation	Nursing Forum	2021	US	To determine the attitudes of nursing students toward interprofessional education (IPE) and teamwork after participating in a lane training mass casualty simulation.	Survey	Nursing + Athletic Training + Homeland Security Studies	Mixture		6 scenarios- 3 focused specifically on emergency care skills (Man Falls off Ladder, Overheated Runner, Bike and Car Accident). 3 had a greater focus on triage and mass casualty (house contamination/bioterrorism, explosion at a fertilizer plant, camping chaos post-tornado)	There was a small group debrief after each simulation before lane change. Then at the end of the seesion there was a debrief with all groups.	Most enhanced skills included communication (93.55%), situation assessment (90.32%), teamwork (90.32%), patient assessment (83.87%), and critical thinking (80.67%). Open-ended qualitative analysis revealed five themes: self-awareness, confidence, valuing teamwork, role conflict, and communication. Students reflected on strengths, weaknesses, teamwork experiences, personal and professional learnings	62% reported the simulation above average or higher for learning about roles in a mass casualty situation.	This study supports the use of the lane training model for teaching emergency care, triage, and mass casualty procedures in an IPE environment.	logistical issues, Time constraints,	IP faculty collaboration, simulation resources (mannequins and volunteers), outdoor setting, debriefing process.	Co-designed.		
6	Breckwoldt	Operating room technician trainees teach medical students - An inter-professional peer teaching approach for infection prevention strategies in the operation room	Antimicrobial Resistance & Infection Control volume	2019	UK	The aim of this study was to design and evaluate an inter-professional peer-teaching module for operating room technician trainees (ORTT) and medical students (MDS) to promote infection prevention and control (IPC) in the operating room (OR).The study aims to assess the feasibility, learning objectives, and educational quality of the module, as well as its potential to foster mutual understanding and patient safety among the student groups.	Descriptive study	Medicine + Operating room technician	Other	To teach the MDS IPC behaviour in the OR by ORTT. ORRT also aimed to improve teaching skills.	ORTT teaching MDS in a simulated OR setting with four posts: entering OR, surgical hand disinfection, dressing up for surgery and preparing a surgical field, and debriefing.	facilitated by educational supervisor, focused on IP exchange	Questionaires: Both ORTT and MDS rated the module highly positive, especially on the aspects of inter-professional exchange, patient safety, and teaching quality. Free text comments: MDS mostly gave positive feedback and suggestions for improvement, while ORTT were more self-critical and reflected on their teaching performance.	Observer field notes: The observers reported that the module stimulated lively discussions, mutual respect, and insight into the other profession's perspective. They also noted some challenges and areas for further development	The authors acknowledged that the study was mainly descriptive and did not provide evidence of actual learning outcomes or impact on patient care.	n/a	Meaningful Learning objectives, Early IPE training in their training,, Near-peer teaching .	Co-designed.		
7	Hardisty	Are students accepting of an all-day conference delivered model of Interprofessional Learning (IPL) on Antimicrobial Stewardship and Patient Safety?	International Journal of Pharmacy Practice	2017	Netherlands	The aim of this study is to determine the students' experience of an all-day conference style event used to deliver a key topic for all healthcare undergraduates surrounding principles of antimicrobial stewardship and patient safety.	survey	Medicine + pharmacy	Mixture	Not noted	WS1. Sepsis case using high fidelty sim. WS2. choosing right antibiotic focusing on prescrbibing in UTI's and meningits. WS3. A patient safety signiicant event audit.	Not noted	>80% of students felt more confident working IP= understood roles, improved communication between professionals and understanding.			not noted	not noted	Co-designed		

8	Reising	Psychometric Testing of a Simulation Rubric for Measuring Interprofessional Communication.	Nursing education perspectives	2015	US	The aim of this study was to establish psychometric testing of the Indiana University Simulation Integration Rubric (IUSIR), a tool for measuring interprofessional communication in simulations.	Quantitative study	Medicine + Nursing	Mixture	1) provide an opportunity to practice and refine team communication skills; 2) improve the application of theoretical skills to real practice.	IPE sim activties took place over 2 years, sim scenarios ranged from 15 to 20 min	The debriefing was conducted right after the scenario by the lead nurse and medical faculty members. student feedback was based on the **IUSIR** ratings and the scenario-specific procedure rubric.		Improved teamwork and communication skills among nursing and medical student. Increased confidence and competence in interprofessional collaboration and patient care. Enhanced understanding of roles and responsibilities of different health professions.		Scheduling and logistics, Faculty development and support, Student engagement and preparation, Tool (IUSIR) refinement and testing,	nursing and medical faculty worked together to create and deliver the simulation scenarios, Student engagement, simulation center	Co-designed	this study was focussed on testing a tool used to asses/measure interprofessional communication competencies in students during IPE simulation.
9	Edwards	Effective interprofessional simulation training for medical and midwifery students.	BMJ simulation & technology enhanced learning	2015	UK	The aim of this study was to implement and evaluate a one-day interprofessional training module for medical and midwifery students.	Prospective Review	medicine + Midwifery	Mixture	Managing obstetric and generic emergencies, such as sepsis, maternal collapse, postpartum haemorrhage and shoulder dystocia.	two sessions: 1. was lectures, small group teaching and clinical skills demonstration for all clinical subjects, together with hands-on practice using manikins (for shoulder dystocia and basic life support). 2. was rotation of four simulated emergency scenarios using high technology and low-technology simulators (SimMom, PROMPT Birthing Simulator, MamaNatalie, ResusciAnne and patient actors)	Structured oral debrief using observer checklists which focus on clinical tasks, teamwork and communication.	No negative comments were identified from students.	Students enjoyed training together, and that this IPT was associated with significant improvement in attitudes to IP working in parallel with significant improvements in factual knowledg		logistical challenges, difficulties recruiting students from multiple faculites, conflicting timetables, separate training campuses	Equal commitment to IP training = overcome this. faculty members had experience in delivering IP obstetric emergency training for qualified staff using simulation and structured debrief. Balanced numbers pf students = avoid professional domminance	co-designed	this study used robustly validated assessment tools designed specifically for the evaluation of IPT.
10	Wada	Engaging a Community Chaplaincy Resource for Interprofessional Health Care Provider Training in Facilitating Family Decision Making for Children at End-of-Life.	Hawai'i journal of medicine & public health : a journal of Asia Pacific Medicine & Public Health	2019	US	The aim of this study is to describe a simulation-based interprofessional education program that involves chaplaincy residents, pediatric residents, nursing students, and social work students in learning how to facilitate family decision making for children at end-of-life	Qualitative study	Medicine + Nursing + Social work (PG) + chaplincy (PG)	Simulated participant	goal of the exercise centered on learning the professional roles and contributions of the interprofessional team involved in end-of-life care planning	Clinical scenario in the PIPP simulation exercise centered on a 3 ½-year-old boy involved in an accidental near-drowning, where there was respiratory/cardiac arrest and aspiration	There was 1 directly after the first part of the sim session which was structured debriefing led by the facilitatorsand then a final debriefing also included a feedback session with the HealthCAST actor, who portrayed the parent of the dying pediatric patient.	feedback regarding teamwork and the contribution of chaplaincy was positive, and expressed the desire for more interprofessional experiences that included chaplaincy residents. Learners valued working as interprofessional team members in these difficult situations		PIPP is currently in its third year, and data and thematic analyses of quantitative and qualitative survey results are ongoing.	N/A	callabortaion between faculties and ministery. State of the art sim centre. collaborative effort between SONDH and the UHM Department of Theatre and Dance. The training of the actors with an "emotional trajectory" was noted be important. Also that the actros particapted in the debrief feeding back how they felt as parents of the child to the particapnats.	Co-designed.	this examines the inclusion in a chaplincey role in IPE sim in breaking of bad news. Intering points about actors.
11	Pederson	Attitudes towards interprofessionalism among midwife students after hybrid-simulation: A prospective cohort study	NURSE EDUCATION TODAY 2021	2021	Switxerland	The aim of this study was to determine the benefit of a hybrid interprofessional simulation on interprofessional attitudes of student midwives, as measured by the German-Interprofessional Assessment Scale (G-IPAS), and whether any beneficial effects were sustained over time.	Prospective Review	Midwifery + Anaesthesia (PG)	Simulated participant	Not documented- but; each scenario had pre-defined learning outcomes and a guided script, the scenarios had slight variations and were not standardised	a woman in labour	instructor-led video-assisted debriefing took place with the entire group. Debriefings reflected the medical processes involved with the cases, but focused specifically on human factors like leadership, teamwork, communication and mutual collaboration.	The simulation activity improved the interprofessional attitudes of midwife students immediately after the simulation, as measured by the G-IPAS questionnaire.	The improvement was not sustained after three months, but the simulation group still had higher interprofessional attitudes than the control group without simulation.		not mentioned	Interdisciplinary facilitators-instructors for teaching and debriefing	co-designed	Immediately after the simulation and debriefing, the participants completed their second G-IPAS ("after"). Here the students rated their interprofessional attitudes at the moment after simulation. The follow-up assessment used the same G-IPAS and was filled in three months later. The follow-up was closed in September 2017.
12	Mcquown	Creation and Implementation of a Large-Scale Geriatric Interprofessional Education Experience.	Current gerontology and geriatrics research	2020	US	Aim of this study was to evaluate the number and type of students who attended a pilot and 10 subsequent educational sessions.	Quantitative study	Medicine + Nursing + Pharmacy + Physiotherapy + Social work	Other	not a simulation of a patient encounter, but a simulation of an IP team meeting.	IP team meeting simulation session, the students began in separate professional huddles.real case with patient identifiers removed was provided by the hospital geriatric team and used by the facilitators to create a patient narrative. e students were then divided into IP teams. Each IP team consisted of 8–10 students representing different professions. In the IP teams, students presented the assessment findings gathered during the huddles. Students were tasked with developing an evidence-based plan of care based on profession-specific assessments and effective team communication.	Facilitators led post-simulation debriefings immediately after the simulation to reflect on the team's priorities of care, team communication, and decision-making.	Students benefited from geriatric specific education aswell as practice working with interprofessional teams during the simulations. Students typically report enhanced realism and immediate feedback provided from standardized patients in medical simulation in comparison to more traditiona lmethodologies. students were able to learn from practitioners who actually function regularly on IP teams	close-to-reality exercise without compromising patient or student safety.		Scheduling students from different disciplines and educational paths seems to be a universal	Grant funding, PDSA model	Co-designed	specific to this study: students simulate an interprofessional team meeting rather than just a patient care scenario was unique to our program and can be used as amodel for others
13	Davis	Health Care Professional Students' Perceptions of Teamwork and Roles After an Interprofessional Critical Care Simulation	Dimensions of critical care nursing	2021	US	The aim of this study was to determine how a simulation-enhanced interprofessional education (Sim-IPE) teaching strategy fostered communication and interdisciplinary awareness between students from multiple disciplines.	Descriptive study	Nursing + Paramedics + Radiography + surgical technology	Simulated participant	4 different states pf simulation; The overarching purpose was to provide a realistic critical care simulation for students from different professions to learn about one another's roles and collaborate during the scenario.	Rugby field, Ambulance, ED, OR, Medical-surgical unit. Paitent was transferred through these 4 states- IPE different students for each state	simulationists who observed the entire simulation debriefed the experience	increased the students' perceptions of teamwork, roles/responsibilities, and patient outcomes across different health care disciplines. students who had more experience in the health care field had higher perceptions of teamwork and roles/responsibilities than those who had less or no experience.		The value of previous exposure to health care for promoting interprofession al collaboration warrants further study.	not mentioned	Coordinating the schedules of six faculty members and five simulation team members. Finding a mutually agreed-upon date to host the event one semester in advance. Developing a multistate scenario that was suitable for differentdisciplines and learning levels. Evaluating the learning outcomes and the business case forsimulation as an improvement technique. Establishing the connection between the simulation and improvement fields, both in practice and in scholarship.	co-designed	
14	Sizemore	Interdisciplinary Education Apartment Simulation (IDEAS) Project: An Interdisciplinary Simulation for Transitional Home Care	MedEdPORTAL : the journal of teaching and learning resources	2021	US	The aim of this study was to create and evaluate an interprofessional simulation activity for home-based care	Survey	Medicine + Nursing + Pharmacy + Physiotherapy + Occupational therapy + Dentistry	Simulated participant	Describe the roles and responsibilities of aninterprofessional team with regards to home-based care(HBC).2. Develop an effective care plan using an interprofessionalteam approach for transitions from acute, hospital-basedcare to the home setting.3. Demonstrate the use of effective communication amongdifferent members of an interprofessional team in HBC.	posthospital discharge home visit using a team of learner	debriefing focused on the interprofessional aspects of the encounter including professional roles and responsibilities,teamwork, and IPE communications. 30-60 mins and was done using open ended questions and a fe debriefing techniques	Increases in self-reportedconfidence in their ability to promote communication as amember of an IPE team understanding of the abilities and contributions of other team members and to develop an effectivecare plan with other health care professionals			Resource intesnity, scheduling issues, lack of untergratedIPE cirriculum - sim was not madatory or graded knock effect of particaotion and effort	Centralised IPE and simulation prgrammes, Faculty engagement and recoginition, Student volunteeerism and motivation, Simulated apartment and standarized patient.	co-designed	

15	Kumar	Interprofessional Simulation-Based Education for Medical and Midwifery Students : A Qualitative Study	CLINICAL SIMULATION IN NURSING	2017	Australia		Qualitative study	Medicine + Midwifery	Mixture	developing respect and a supportive relationship " of equals"with eachother.	1. Speculum examination, bimanual examination, and performing a pap smear, 2. Vaginal examination and assessment in labour, and 3. Conducting a normal vaginal birth with estimation of blood loss		key themes reported: Medical students reported the importance of ''learning by doing'' through simulation. Midwifery students was focused on ''relationship of power'' compared with the other discipline.	positive influence on the attitude of medical and midwifery students, inspite of the disparity in their background knowledge and experience	medical and midwifery students were keen to have an understanding of the other profession's curriculum. Both were open to curricular changes with a view to integrate medical and midwifery curricula, where relevant.	scheduling difficulites, uneveen backgrounf knowledge, lack of follow up	Different professional satff owkring togther, student engamenet, simulation design= realistic and releveant.	co-designed		
16	Yu	Simulation in the medical undergraduate curriculum to promote interprofessional collaboration for acute care: a systematic review.	BMJ simulation & technology enhanced learning	2016	England	review sought to systematically collect and summarise published peer-reviewed literature describing undergraduate or prelicensure, interprofessional, simulation-based educational interventions aimed at improving the knowledge and skills required for assessment and management of acute medical conditions	Systematic review	N/A												This study found that simulation-based IPE can help medical and other health professional students to acquire the skills and attitudes needed to work as effective clinical teams in acute care settings. SBIPE= diverse and cpmplex= careful design and delivery. SBIPE= improve students outcomes such as self-reported understanding of IP rooles, teamwork principles, patient advocacy and clinical skills. SB-IPE = improves team outcomes - team process, team effectiveness, error rate, and response time. this paper also found that SB-IPE can benefit from use of theoretical frameworks, standardised reporting checklists, and interprofessional faculty facilitators.
17	Lippe	Palliative Care Educational Interventions for Prelicensure Health-Care Students: An Integrative Review	AMERICAN JOURNAL OF HOSPICE & PALLIATIVE MEDICINE	2018	US	purpose of this integrative review was to critically evaluate the impact of palliative or EOL care interventional studies on learning outcomes for prelicensure health-care students.	Scoping review	N/A											this is a scooping review	Interprofessional education is needed for palliative care. Interprofessional education is not well implemented or evaluated. Interprofessional education should focus on less explored outcomes and populations. (e.g examine the effects of interprofessional education on outcomes such as communication, competence, and confidence, as well as on specific populations, such as pediatric patients or patients from diverse cultural backgrounds).
18	Yang	Impact of an Interprofessional Surgical Skills Workshop on Undergraduate Medical and Nursing Student Interest in a Career in Surgery: A Thematic Analysis	Journal of Surgical Education	2021	Australia	aim of this qualitative the matic analysis is to explore: (1) medical and nursing student's influences on their perspectives on surgery, and (2) the impact of a novel, interprofessional, simula tion-based workshop on medical and nursing student interest in surgery as a career	Qualitative study	Medicine + Nursing	Task manikin		workshop - skills-station circuit - 3 stations; 1. Hand ties. 2. suturing. 3. female catherization 40 mins @ each	No debrief mentioned however- facilitators provided feedback on indivudal and group level for all taught skills and actively encourged students to leanr from and with each other IPL and personal reflection was encouraged. = multidsiciplnery fcilitators.	pre- and postworkshop paper based survey was completed by students before and immediately after the workshop. Assess individual student's changes in perception of surgery after the workshop.	Simulated Practice of Technical Skills: Both medical and nursing students reported increased confidence and enthusiasm to practice technical skills taught during the workshop. Simulated practice led to a decrease in intimidation towards attending theatre and increased proactive involvement during surgical rotations. Some students expressed that simulated practice made a career in surgery seem more achievable. Exposure to Nontechnical Aspects of Surgery: Nursing students found the interprofessional nature of the workshop, reflecting the collaboration between surgeons and nurses, to be novel and interesting. This interprofessional setting increased interest in surgery as a career among nursing students, medical students did		Discrepancy between medical and nursing student NUMBERS. Scheduling conflicts . Timing of the workshop (WS was held before the nursing students had their periopertaive placement = limited exposure)	Multidisciplinary facilitators. True reflection of the IP nature of surgery (IPC). Novelty of IPE: Nursing students in particular responded to the interprofessional aspect of the workshop and commented on how awareness of this positive non-technical aspect of surgery increased their interest in perioperative nursing	Co-designed	Point; Behavior of both surgeons and operating theatre nursing staff influences medical students' experiences, leading to decreased engagement and reduced interest in surgery. Pre workshop perceptions intering Inclusive Mentors and Role Models: Inclusivity enhances students' comfort levels, encourages active participation, and inspires them to consider surgery as a career.Nursing students find assisting in theatre to be an excellent learning opportunity, which enhances their perceptions of perioperative nursing. Learning Through	
19	Southall	Fostering Undergraduate Medicine, Nursing, and Pharmacy Students' Readiness for Interprofessional Learning Using High Fidelity Simulation	Cureus	2021	US	to explore student's readiness for interprofessional learning and determine whether participation in high fidelity interprofessional education resulted in higher levels of readiness for interprofessional learning.	Mixed methodology	Medicine + Nursing + Pharmacy	Full body manikin	To enhance students' understanding of the clinical management of an adult patient experiencing acute anaphylaxis. To foster interprofessional teamwork and collaboration IPE	simulation was based on managing the care of a patient experiencing acute anaphylaxis. To assess students' readiness for interprofessional learning and the impact of high fidelity simulation on their attitudes and perceptions	30-minute debriefing session it occurred in a classroom setting and was part of the interventional program. The purpose was to provide feedback and reflection on the simulation experience.	Post sim significant improvement in positive attitudes towards teamwork and collaboration, reflecting a better understanding of clinical problems, enhanced communication skills, increased understanding of professional limitations, more trust and respect in the team, and the benefits of interprofessional learning opportunities		The findings from this study suggest that all of the participants were ready for interprofessional learning and would welcome the opportunity to share more opportunities for interprofessional learning through lectures, tutorials, or workshops with other healthcare students.	difficult task of coordinating student schedules to learn together	not menioned	co-designed		
20	Reising	Team Communication Influence on Procedure Performance: Findings From Interprofessional Simulations with Nursing and Medical Students	Nursing education perspectives	2017	US	The aim of this study is to determine whether interprofessional team communication is related to team performance in a simulation with nursing and medical students.	Retrospective Study	Medicine + Nursing	Other	students were rated on: individualcommunication, team communication, and procedure performance.	students on IP teams for 2 years range of IP activties eg sim. 1) an asthma scenario. 2) an advanced scenario of diagnostic reasoning and Advanced Cardiac Life Support	A 10-minute session that followed immediately after the simulation, used the IUSIR and procedure scoring tools to guide the discussion and feedback.	results of this study demonstrate that improved interprofessional team communication was related to improved procedural perfor mance= impoved paitent care.	Simulation is an effective means to recreate practice situations that promote the development of team communication skills in order to improve patient care.		not mentioned	not menioned	co-designed		

21	Paige	Brick in the wall? Linking quality of debriefing to participant learning in team training of interprofessional students	BMJ Simulation and Technology Enhanced Learning	2021	UK	the quality of debriefing in using simulation- based training in team training correlated with the degree of learning of participants.	Retrospective Study	Medicine + Nursing	Full body manikin	The simulation aimed to improve the students' teamwork and communication skills as well as their clinical knowledge and skills related to trauma care.	The simulation used a dual scenario design, in which each student team underwent a prebrief, a first scenario, an immediate after-action debrief, a second scenario, and another immediate after-action debrief. The simulation was integrated into the mandatory curriculum of the students' surgery rotation and intensive care course	one to three instructors leading a debrief at one time. None of the instructors had undergone a formalised course in debriefing training. Each debrief would begin with an investigation of student participants' emotional response to the scenario (ie, How did that feel?). It would then analyse key actions to identify performance gaps and address learning objectives to help fill those gaps. Finally, it would finish with a summary of teaching points with an elicitation of student commitment to work on one item taught.	The quality of prebriefs and debriefs in interprofessional education using simulation-based training did not have an effect on the degree of learning of participants as measured by improvement in their team-based performance during simulation-based training sessions.	debriefs have a powerful influence on team function and performance	Designing scenarios for multiple learners. Anticipating learner responses to simulation events. Measuring non-technical teamwork skills. Providing effective feedback to an interprofessional audience (tailored debriefs to learning needs of students/profession etc)	simulation activity was integrated into the mandatory courses of the medical and nursing students, ensuring their participation and alignment with learning objectives. faculty teams received training and feedback on how to conduct effective prebriefs and debriefs, as well as how to use the simulation equipment and tools. high-fidelity manikins, equipment, video recording, and assessment tools.	co-designed	highlights importance of debrief	
22	Wang	Implementation and evaluation of an interprofessional simulation-based education program for undergraduate nursing students in operating room nursing education: a randomized controlled trial	BMC medical education	2015	China	implement an interprofessional simulation-based education program for nursing students and evaluate the influence of this program on nursing students' attitudes toward interprofessional education and knowledge about operating room nursing.	Randomised control trial	Medicine + Nursing		To enhance nursing students' knowledge and skills in operating room nursing. improve nursing students' attitudes toward interprofessional education and collaboration. provide nursing students with an opportunity to apply learned concepts and skills in a realistic clinical setting with medical students.	appendectomy, splenectomy, and small bowel resection and anastomosis. 3hrs and occurred weekly over 2 week period. nursing students acted as scrub or circulating nurses, and medical students acted as surgeons.	Not mentioned	improved nursing students' attitudes toward interprofessional learning and knowledge about operating room nursing. Nursing students in the IPSE group showed more positive responses to four questions in the Readiness for Interprofessional Learning Scale, reflecting a more positive attitude toward teamwork and collaboration and professional identity. Nursing students in the IPSE group also showed significantly higher scores on the knowledge questionnaire, indicating a higher level of knowledge about infection control, patient safety, quality assurance, and professional accountability in operating room nursing.	Nursing students' qualitative feedback revealed four themes: communication with medical students, role awareness, a better way of learning, and future IPSE.	logistic constraints in the delivery of this IPSE program, as synchronizing sessions for small groups of students in different professions	authors mentioned that the IPSE program was supported by the Tianjin Medical University faculty members, who ensured that the simulation scenarios incorporated aspects and problems important across the spectrum of professions.	co-designed	ONLY talks abouts nursing students results/impacts. Also this simulation was carried out on animal models	
23	Onan	A review of simulation-enhanced, team-based cardiopulmonary resuscitation training for undergraduate students	Nurse education in practice	2017	Turkey	The aim of the study is to review and synthesize published studies that address the primary question "What are the features and effectiveness of educational interventions related to simulation-enhanced, team-based cardiopulmonary resuscitation training?"	Systematic review	N/A										Results talk abou simulation but not IPE	Simulation-enhanced, team-based CPR training for undergraduate students is effective in improving their satisfaction, knowledge, skills, and retention of CPR performance. Simulation-based learning has more impact on skill acquisition than knowledge acquisition, and the effect is more evident in long-term follow-up tests. Simulation-based learning can also enhance students' self-confidence, attitudes, and perceptions of CPR and teamwork. There is a lack of studies that investigate the transfer of simulation learning to clinical practice and its effect on patient outcomes
24	Marion-Matins	Interprofessional simulation effects for healthcare students: A systematic review and meta-analysis	Nurse education today	2020	Brazil	systematic review of studies describing the effects of (IPE) on collaborative competence using simulated-based training of undergraduate healthcare students (results= participants, protocols, scenarios, validated tools, collaborative competencies and primary outcomes)	Systematic review	N/A											This paper found that IPE sim with HCP students had positive effect on improving their collaborative competencies, such as communication, teamwork, roles and responsibilities, and patient-centered care. Limitations/challenges identified = risk of bias, the heterogeneity of the studies, the lack of clinical trials, and the need for more research on primary care scenarios and long-term outcomes. Concluded with: IPE sim = useful and effective methodology that should be added to the undergraduate curriculum of health care students.
25	Aldriwesh	Undergraduate-level teaching and learning approaches for interprofessional education in the health professions: a systematic review	BMC medical education	2022	UK	The purpose of the study was to conduct a systematic review to establish how IPE has been implemented in university-based undergraduate curricula, focusing on the teaching and learning approaches.	Systematic review	N/A											IPE teaching and learning approaches: A combination of at least two teaching and learning approaches was used to deliver IPE in undergraduate curricula for healthcare professions. Simulation-based education, e-learning, and problem-based learning were the most prevalent approaches. IPE participants and settings: Nursing, medicine, and physiotherapy were the most frequent healthcare professions that participated in IPE programs. Most IPE programs were implemented in university-based settings, and some were integrated in the existing undergraduate curricula. identifed a lack of IPEin the middle east region.
26	Herath	A comparative study of interprofessional education in global health care A systematic review	MEDICINE	2017	China	The aim of this study was to examine the incidences of interprofessional education (IPE) and summarize the main features of IPE programs in undergraduate and postgraduate education in developed and developing countries	Systematic review	N/A											IPE programs vary substantially across different countries, with developed countries having more IPE initiatives than developing countries (not enough to meet global health targets) . IPE programs are mostly at the undergraduate level, and involve mainly nursing, medicine, pharmacy, and dentistry disciplines. IPE programs face several challenges and barriers in their implementation, such as curriculum, leadership, resources, stereotypes, and accreditation issues. IPE programs need to be aligned with the practice needs and health outcomes, and require more evidence-based evaluation and policy support.

27	Dinh	Interprofessional disaster exercises for undergraduate nursing students: a scoping review	JBI Evidence Synthesis	2023	US	The aim of this scoping review was to report on the planning and implementation of a disaster exercise for undergraduate nursing students, which included the participation of other health students, allied health students, or professionals, for the purpose of preparing them to respond to a disaster	Scoping review	N/A			review found a small number of studies describing the presence of a multidisciplinary team in disaster exercises, primarily for nursing students= not a priority in nursing cirricula. IPE opputunity= enhanced understanding of interprofessional teamwork, interprofessional communication, clinical skills acquisition, and effective teamwork in a disaster. oppurtunity for students to observe other professions = refelect and practice. most included debriefing. majority of the exercises were conducted to meet specific learning objectives. All papers= IPE exercises = logistcally challenging== adequate training for volunteer patients; the need for better briefing of participants; adequate resourcing and consumables to authentically represent real-life scenarios; ensuring that adequate time was allocated for conducting the exercises, as well as the planning beforehand; and allowing for effective debriefing after the disaster exercise.
28	Khan	Currently Available Tools and Teaching Strategies for the Interprofessional Education of Students in Health Professions: Literature review.	Sultan Qaboos University medical journal	2016		targets IPE issues involving undergraduate health profession students and highlights various approaches in different regions	Literature Review	N/A			Importance of IPE: IPE is crucial for developing an efficient and effective healthcare workforce by fostering interprofessional collaboration, improving patient care quality, and addressing the complexities and fragmentation in healthcare delivery.<br>Levels of Interaction: IPE facilitates three levels of interaction - communication, mutual respect/trust, and teamwork among healthcare professionals, leading to better adoption of Interprofessional Collaboration (IPC) in healthcare settings.<br>Communication, Mutual Respect, and Trust: IPE activities help in developing and demonstrating communication skills, fostering mutual respect and trust among healthcare professionals, and increasing awareness of each other's roles and limitations.<br>Teamwork: IPE enhances flexibility, adaptability, and teamwork skills among students, preparing them for collaborative practice in real-world healthcare settings.<br>Implementation in Curriculum: Key concepts for implementing IPE in medical undergraduate curriculum include early introduction, utilization of various instructional methods, and incorporation into existing courses. Longitudinal curriculum design with interprofessional events embedded in practical learning experiences is recommended.<br>Assessing Impact: Assessment of IPE impact includes administering surveys, objective-structured clinical examinations, and providing formative feedback to students. Positive changes observed include improved opinions regarding IPC, enhanced professional confidence, and better team dynamics.<br>Challenges to Implementation: Challenges in implementing IPE include lack of standardized assessment instruments, facilities, trained personnel, and resistance among faculty members. There's also a need for agreement on crucial learning outcomes and more models of best practice.<br>Inconclusive Evidence on Effectiveness: Studies show inconclusive evidence on
29	Lackie	Creating psychological safety in interprofessional simulation for health professional learners: a scoping review of the barriers and enablers	Journal of interprofessional care	2023	Canada	The aim of this study is to understand the barriers and enablers of psychological safety within interprofessional simulation-based education	Scoping review	N/A			PS is influenced by many factors; sim design, facilitation. Hierarchy, observation and uncertainity. PS= enhanced by pre-briefing-debriefing, no blame culture, structured evidence-based sim, skilled facilititaion= trust, respect and openness. PS= hindered by power dynmamics, fear of making mistakes, lack of clarity, and varibales in sim and debrief quailty. PS found to be important gor IPL = students to participate fully, share perspectives, challenge assumptions and learn form each other. PS in debrief NB (trained facilitators in debrief) this paper also highlights the lack of common vocab for this stopic in education and research highlighting the limitation of this scooping review as its hard to gauge if everything has been included due to the lack of standarised vocab.
30	vanWyk	Simulation as an educational strategy to deliver interprofessional education	AFRICAN JOURNAL OF HEALTH PROFESSIONS EDUCATION	2020	Africa	The aim of this study is to identify current IPE methods used in undergraduate programmes in the Faculty of Health Sciences at the University of the Free State, Bloemfontein, South Africa, and to determine the opinions of module leaders on using simulation as a particular IPE teaching strategy.	Quantitative study		Nutrition + Dietetics + Occupational therapy + Physiotherapy + Optometry + Nursing + Medicine	The main advantages of using simulation for IPE were improved role clarification, empathy and respect for other professions, and a safe learning environment for students and patients. The main challenges of using simulation for IPE were scheduling, logistical and resource issues, and the need for a paradigm shift among staff and students. in this university A three-phase longitudinal approach was proposed to enhance IPE in the undergraduate programmes, consisting of didactic, simulation and community-based learning experiences.	
31	Granheim	The use of interprofessional learning and simulation in undergraduate nursing programs to address interprofessional communication and collaboration: An integrative review of the literature	Nurse education toda	2018	Australia	To identify how simulation and IPL are used togther in undergradyuate nursing prograams and undertaken in schools of nursing to adress IPC and collaboration	Literature Review	N/A		Only adresses Nurses.	4 key themes emerged- collaboration (IPL= mproves worked better togther after sim), learning in practice (IPL sim = opputunity to practice and improve skills, make mistakes without impact on patients), understanding roles (encouraage students to engage with eachother+ learn from each other, conflict management improved after sim) and communication (combining IPL and Sim = useful communication enhanced). The implementation of IPL and simulation in undergraduate nursing programs is challenging and variable, depending on factors such as timetabling, curriculum design, faculty support, and student readiness.
32	Velasquez	Interprofessional communication in medical simulation: findings from a scoping review and implications for academic medicine	BMC medical education	2022	UK	The aim of this study is to describe the characteristics of simulation-based interprofessional education (IPE) activities involving undergraduate medical students in a simulation center that focused on interprofessional communication.	Scoping review	N/A			Majority of sims used High fidelty (76%). Standasied patients used in 71%. 29% used manikns only. 33% used hybrid activites (more than 1 resource). Medicas +nurses most common. significant variability in terms of health-related topics taught and instruction methods employed during simulation IPE activities focused on interprofessional communication in simulation centers. Standardized patients provide a clear benefit over role play in an authentic, yet 'safe' first experience in clinical interactions, profess feedback from paitents perspective. costs assocaited with SP = limits them even with all the benefits assocaited for IPC. identifed significant variability in the assessment of simulation IPE activities majority focussing on attitudes/perceptions.

33	Havyer	Addressing the Interprofessional Collaboration Competencies of the Association of American Medical Colleges: A Systematic Review of Assessment Instruments in Undergraduate Medical Education	Academic medicine : journal of the Association of American Medical Colleges	2016	US	To summarize characteristics and validity evidence of tools that assess teamwork in undergraduate medical education(UME), and provide recommendations for addressing the interprofessional collaboration competencies of the Association of American Medical Colleges (AAMC).	Systematic review	N/A											results looking at tools for assessment based on america colleges soley.	There are 64 teamwork assessment tools used in undergraduate medical education, but only 7 of them measure teamwork in clinical settings. Most of the tools (47; 73%) assess interprofessional teamwork, but few (18; 26%) have been tested in multi-institutional studies. None of the studies examined the impact of teamwork assessment on patient outcomes. Four tools were recommended to assess the AAMC's teamwork competencies: the Collaborative Healthcare Interdisciplinary Relationship Planning Scale, the Readiness for Interprofessional Learning Scale, the Communication and Teamwork Skills assessment, and the Teamwork Mini-Clinical Evaluation Exercise.
34	Burke	Undergraduate Health Professional Students' Team Communication in Simulated Emergency Settings: A Scoping Review	CLINICAL SIMULATION IN NURSING	2021	Australia	The aim of this study is to explore what is known about undergraduate health professionals team communication in simulated emergency settings.	Scoping review	N/A												RQ= "What is known about the factors that influence undergraduate health professionals team communication in simulated emergency settings?". It focussed on the communciation side of SIM. use of video recordings for retrospective rating of participant behaviour strengthened the approach- aloowed students to reflect on thier actions they made, ability to scrutinise an action at time of action and make dsecion based on sim and expertise. TeamSTEPPS was the only such program used to facilitate learning of team skills. Patient safety and human factors were identified and discussed as a main motivation for the study. most of the studies, teamwork was reviewed even though the aim or research question was to specifically measure communication. measured teamwork and communication via raters considered this more accurate and reliable than self- assessment. others considered that self-assessment tools enable users to gain insights into their own thinking. some didnt have raters and some valued the students voice more. combing students from different learning experince= experince different ways of viewing and responding to situations. performance in undergraduyates lacked (clinical skills, teamwork and situation awareness). Barriers- lack of knowledge, lack of experince, limited leadership skills and uncertainty -ve impact learning outxcomes.
35	Smith	Qualitative Analysis of Student Physical Therapist Reflective Writing: Does an Interprofessional Discharge Planning Simulation Increase their Understanding of the Role they play in Discharge Planning?	INTERNET JOURNAL OF ALLIED HEALTH SCIENCES AND PRACTICE	2020	US	The aim of this study was to determine, by using qualitative analysis of reflective writing, if an interprofessional discharge planning simulation will increase students' understanding of the role of physical therapy in the discharge planning proces.	Qualitative study	Nursing + Social work + Physical therapy + Doctor of nursing	Simulated participant		Students met paitent and his sister and a meeting of 'discharge occurred' facilitators were in room observing quietly.	students had a ten-minute pre-briefing meeting to discuss the case and complete a worksheet to help guide them in the meeting. After sim meeting simulated participants left the room and each of the facilitators led a thirty minute debriefing.	recorded and placed on the e-learning platform for students to review and allow for self-reflection. Only PT students fillled out relfection paper. (they used reflective writing).	The simulation helped the DPT students to identify the main discharge issue related to patient safety and fall risk, and to communicate their recommendations with the interprofessional team. The simulation also enhanced the DPT students' understanding of their own role and the roles of other professions in the discharge planning process, and highlighted the need for educating other team members about the PT's role. The simulation exposed some gaps in knowledge and skills among the DPT students, such as insurance regulations, coverage issues, and communication strategies for difficult situations.The simulation was a valuable learning experience that could be improved by providing more information and resources to the students, repeating the simulation at different points in [illegible]		Recruitment of simulated participants: It was difficult to find enough older adults who were willing and able to play the role of the patient and the sibling. Scheduling of students: It was challenging to coordinate the schedules of students from different programs and ensure their attendance and participation. Preparation of students: Some students did not review the patient chart or the learning objectives prior to the simulation, which affected their performance and engagement. Evaluation of students: It was hard to measure the impact of the simulation on the students' knowledge, skills, and attitudes, as there was no standardized assessment tool or outcome measure.	Using standards of best practice for simulation-enhanced interprofessional education and simulated participant training. Providing information and resources to the students prior to the simulation via a web-based learning platform. This helped the students to prepare for the simulation and review the patient chart and the discharge planning process. Having facilitators from different disciplines to guide the pre-briefing, the simulation, and the debriefing.	this was designed by the researchers and implemented	Only adresses PT students reflections.	
36	Yu	Evaluation of a modified 16-item Readiness for Interprofessional Learning Scale (RIPLS): Exploratory and confirmatory factor analyses	Journal of interprofessional care	2018	UK	The purpose of this study was two-fold: to establish the presence of a stable subscale structure upholding a modified 16item RIPLS instrument and to assess the psychometric properties of this commonly used tool in order to contribute to an increasing body of literature.	Quantitative study	Medicine + Nursing + Pharmacy	Mixture	goals were to improve participant attitudes, perceptions, and knowledge around interprofessional teamwork in the context of managing acute hospital ward medical problems	WardSIM' is a two-day IPE course developed to prepare undergraduate students for collaborative workplace interprofessional practice		modified RIPLS instrument was distributed at six WardSIM courses						not really relevant. Implications: The modified 16-item RIPLS may be a promising tool for measuring undergraduate students' readiness for interprofessional learning, but it requires further testing and refinement3. The authors recommend caution when using the RIPLS or its modified versions without adequate psychometric evaluation	Not sure how/ information to take from thi study/????
37	Turrentine	Interprofessional training enhances collaboration between nursing and medical students: A pilot study.	Nurse education today	2016	Scotland	The aim of this study is to enhance interprofessional education between nursing students and medical students in a clinical environment, by modeling and practicing the skills needed for interprofessional preoperative geriatric assessment for complex geriatric patients undergoing surgery. The study also evaluates the effectiveness of this educational intervention on students' knowledge, attitudes, and skills related to geriatric assessment and interprofessional [illegible]	Pilot study	Medicine + Nursing	Simulated participant		two-hoursessions in theClinical Skills Laboratory were offered for nursing and medical student pairs to work with clinical mentors patients exhibiting mild dementia, Parkinson's disease, and frailty, respectively.	Not mentioned	students showed significant improvement in their geriatric assessment knowledge and skills after the IPE intervention, students identified some challenges and learning needs, such as scheduling, refining their follow-up questions, and redirecting patients who tend to ramble.	students valued the opportunity to learn from and with each other, and to practice in a clinical setting with supportive mentors. students demonstrated IPE skills such as shared problem-solving, conflict resolution, recognition of patient needs, shared decision making, communication, and transfer of interprofessional learning.	Authors suggested some recommendations for future research, such as expanding the pilot program, refining the evaluation methods, and following up the patients' outcomes.	scheduling difficulties and small sample size.	Providing practice opportunities for the students to work with standardized patients and real patients in nurse/physician pairs, with feedback and guidance from clinical mentors. Using multimedia content such as a video clip of experts performing the geriatric assessment and an online copy of the ACS guidelines to enhance the students' learning and preparation.	Co-designed		

38	Tauscher	Interteam PERINAT - interprofessional team collaboration in undergraduate midwifery and medical education in the context of obstetric emergencies: Presentation of simulation scenarios and empirical evaluation results	GMS journal for medical education	2023	Germany	The aim of this study is to evaluate the effectiveness and feasibility of an interprofessional simulation training program for undergraduate medical and midwifery students in the context of obstetric emergencies.	Descriptive study	Midwifery + Nursing	Mixture	Shoulder Dystocia: Define the term shoulder dystocia and identify the features that lead to the diagnosis. Identify one antepartum and two subpartum risk factors for the presence of shoulder dystocia. Name general and specific measures (e.g., McRoberts maneuver) in the presence of shoulder dystocia and demonstrate them hands-on in the scenario. Postpartum Hemorrhage: Define the term postpartum hemorrhage and identify the features that lead to the diagnosis. Identify risk factors for the development of postpartum hemorrhage. Name immediate obstetrical measures in the presence of postpartum hemorrhage and demonstrate these hands-on in the scenario.	3-hour scenario training was divided into 3 parts: theoretical preparation, the actual scenario training, followed by debriefing and feedback. 15 min: four students each actively participated in the scenario, and four students observed the scenario focusing on professional andteamcommunicationaspects	debriefing and feedback sessions were divided into the following items:1. team self-reflection, 2. feedback from SPs (mother and father roles), 3. observing students, and finally 4. faculty members.	study showed that simulation training can improve communication and teamwork skills in interprofessional obstetric teams, as well as professional skills in emergency situations. study revealed that interprofessional education is underrepresented and needed in undergraduate medical education, especially in obstetrics, where practical experience is limited.			high personnel and time costs, and the difficulty of coordinating different disciplines and curricula as limitations	Interprofessional collaboration project involved faculty members from different disciplines, he project received a grant from the University of Leipzig to promote innovative teaching projects, which helped cover the costs of personnel, equipment, and materials. The use of hybrid simulation with simulated patients and a birthing simulator enabled realistic and immersive training of obstetric emergencies, as well as direct feedback from the patient's perspective. the participants were recruited on a voluntary basis and expressed high interest and satisfaction in the interprofessional simulation training, as well as a desire for more such opportunities in the future.	Co-designed	
39	Visker	Effect of a rapid e-learning module and brief interprofessional simulation event on medical and nursing student collaborative attitudes and behaviors	International journal of nursing education scholarship	2020	Germany	The aim of this study was to determine the effect of a rapid e-learning module and a brief interprofessional simulation event on medical and nursing student collaborative attitudes and behaviors.	Descriptive study	Medicine + Nursing	Mixture	Define the term shoulder dystocia and identify the features that lead to the diagnosis.	a 10-min pre-briefing on their IPE Simulation Event scenario with medical and nursing faculty members. A 20-min long, interprofessional, high-fidelity simulation scenario was performed. nursing student performed a nursing assessment on the simulated patient/manikin, communicated the assessment and recommendations to themedical student, and both collaborated to determine an appropriate plan of care	10-min long debriefing session with medical and nursing faculty immediately followed as a standard part of the event	The study found that both the computer module and the IPE simulation event alone improved interprofessional collaborative attitudes, but the combination of the two did not have a greater effect than the simulation alone. The computer module may be better used as a preparatory activity before the simulation event		The study suggested that educational institutions can benefit from using either the computer module or the IPE simulation event to enhance interprofession al collaborative attitudes among medical and nursing students.	small smaple size of prticpants	simulation centre+ technology	Co-designed	
40	Turkelson	Piloting a Multifaceted Interprofessional Education Program to Improve Physical Therapy and Nursing Students' Communication and Teamwork Skills	Journal of Acute Care Physical Therapy	2018	US	The aim of this study was to examine the effectiveness of a multifaceted strategy including didactic and a standardized patient SBLE on student perceptions of interprofessional teamwork and communication from 3 health care profession	Prospective Review	Physiotherapy + Nursing	Simulated participant	Identify one antepartum and two subpartum risk factors for the presence of shoulder dystocia.	interprofessional handoff communication was identified as a significant due to different professional language. Simulation scenario was based on a patient with multiple sclerosis who was admitted to the hospital after surgical repair of a fractured right humerus secondary to a fall at home- orders included gait evaluation and treatment from physical therapy and discharge instructions from nursing.	One week after the simulation sessions, all teams reconvened in 1 large debriefing session to discuss the video reviews and self-reflections. IPE teams were reunited to share with each other and the larger group their postsimulation reflections. both professions facilitated the large group discussion using the Plus Delta Framework	Using a multifaceted approach allowed students to gain valuable experiential knowledge and practice in a risk-free setting fostering self-confidence and skill development; both of which are essential for safe interprofessional clinical practice. program did not show a significant effect on students' perceptions of interprofessional collaboration, as measured by the IEPS, which could be due to the limitations of the tool or the short duration of the intervention. All students demonstrated a high level of satisfaction with the simulation learning and self-confidence in the skills and knowledge presented in the simulation.		highlighted the need for further research on the best practices and outcomes of interprofession al education, especially in terms of measuring performance, behavior change, and patient outcomes.	The authors mentioned that coordinating the schedules of students from different programs and faculty from different disciplines was challenging and time-consuming. They also had to accommodate the availability of the standardized patients and the simulation center. number of students in each profession was not equal, which resulted in some students participating in multiple simulation sessions or being paired with students from the same profession.		Co-designed	
41	Uslu-Sahan	Interprofessional simulation-based training in gynecologic oncology palliative care for students in the healthcare profession: A comparative randomized controlled trial	Nurse education today	2020	Turkey	The aim of this study was to determine the effectiveness of different simulation methods used for interprofessional training on gynecologic oncology palliative care knowledge, interdisciplinary education perceptions, and teamwork attitudes of health professional students, and to compare these methods	Randomised control trial	Medicine + Nursing + Nutrition-dieticion + Social work	Mixture	Name general and specific measures (e.g., McRoberts maneuver) in the presence of shoulder dystocia and demonstrate them hands-on in the scenario.	scenario= female aged 66 diagnsed with stage 3 ovarian cancer 2 yrs ago, presented to ER a week ago- high amount of acid, visualscale analogue of 8- wants to be disvcharged from hospilat NOW.. P1. Nurse interview and a team meeting with the patient and her relative (10 min). In this part, the nurses perform patient handover; first the nurse and then the team evaluates the patient and her daughter and perform the required palliative care interventions. This part ends after the evaluations and interventions are complete. P2. Team meeting: The patient's general condition is evaluated in the team meeting room and the team decides to discharge her from the hospital. P3. Sharing the discharge decision with the patient and her daughter: The discharge decision is shared and discussed with the patient and her daughter.	not mentioned	results focussed on pallative care knowledge	this study identifed none of the students had particpated in IPE before and 66.7% had not particapted in simulation before. At the time of this study the concept of IPE training was very new in turkey. literature has emphasized that simulation-based interprofessional training in palliative care increases knowledge levels and competencies of students in related subjects. observed that training interventions without simulations have a limited effect. HFS + HS application in interprofessional simulation-based training effectively increased the students gynecologic ontological palliative care knowledge, interdisciplinary education perception, and teamwork attitude.		identification stage that there is no well-established interprofessional training education in Turkey, and students often resist this kind of training. Difficulty - students' intense program and in obtaining permission from the related institutions to conduct the study with students from several disciplines.		Co-designed	
42	Labrague	Interprofessional simulation in undergraduate nursing program: An integrative review	Nurse education today	2018	US	The aim of this study was to systematically appraise and synthesize evidence examining the effects of interprofessional simulation on nursing students' outcomes	Literature Review	N/A		Postpartum Hemorrhage:									revealed five essential themes: interprofessional communication (important outcome- IPE-SB demonstrated a significant increase in their communication skills with patients and other healthcare team members), appreciation of interprofessional team roles (self-explanatory) , interprofessional teamwork or collaboration (prominent theme), self-confidence or self-efficacy (participated in simulation demonstrated an increased levels of confidence in caring for patients with various medical problems such as infections, injuries, and cerebrovascular disease. after IP-SBE, nursing students demonstrated comfort and confidence in managing patients who are giving birth), and positive attitudes or readiness toward interprofessional learning (nursing students who participated in interprofessional simulation reported a significant increase in readiness for interprofessional learning). This review found alot of variation between papers with- timing, duration, and facilitation of the simulation activities varied across studies, which may affect the measurement and generalization of the outcomes.

43	Yang	Introduction of an interprofessional gynaecology surgical skills workshop for undergraduate medical and nursing students	Australian and New Zealand Journal of Obstetrics and Gynaecology	2020	Australia	the aim of this study was to explore students' perceptions of a simulation-based interprofessional gynaeco-logical skills program	Pilot study	Medicine + Nursing	Mixture	Define the term postpartum hemorrhage and identify the features that lead to the diagnosis.	two-hour workshop with simulated, supported practice of common surgical skills using low-maintenance task-trainers.	Verbal individual and group feedback were provided during and immediately after practice. Reflection on procedural aspects and interprofessional interaction was actively encouraged	Results focussed on surgical skills- Both medical and nursing students reported a statistically significant increase in their confidence scores for all four taught skills after the workshop. Most medical and nursing students agreed that their course should provide more practical surgical education, such as the workshop program. IPE results- Confidence in interprofessional behaviours also improved in both cohorts, with medical students showing a significant improvement. Students also reported positive attitudes toward interprofessional education and collaboration.		highlighted the need for more structured and authentic IPE activities in the undergraduate medical and nursing curricula, as well as the challenges of aligning the learning objectives, assessment methods, and accreditation standards of different professions.	The number of nursing students who participated in the workshop was much lower than the number of medical students, due to scheduling conflicts and voluntary enrolment. This limited the generalisability and interprofessional balance of the workshop.		Co-designed	
44	Stewart	Promoting System Thinking and Professionalism Through Simulated Hospital Experiences for Nursing and Clinical Laboratory Science Students	Nursing education perspectives	2023	US	The aim of this study was to explore the effects of an interprofessional simulation laboratory for nursing and clinical laboratory science students on their aspects of professionalism, such as communication, respect, ethics, and accountability	Mixed methodology	Nursing + Clinical laboratory science		Identify risk factors for the development of postpartum hemorrhage.	participated in simulated hospital scenarios as rotating small-group mixed-cohort teams. The teams worked with faculty-developed patient cases with laboratory test results during the two-day seminar in a CLS laboratory. Seminar Procedure 30-minute experience, the students had 10 minutes to review their assigned case and 20 minutes to discuss and answer five case-specific questions.	At the end of the simulation, team members discussed the correct answers provided by the faculty to the case study questions	RQ= whether IPE using peer-to-peer problem-based learning enhances aspects of professionalism, such as communication, mutual respect, excellence in practice, and ethics and accountability. students' scores on the modified Interprofessional Professionalism Assessment increased significantly after the simulation, indicating enhanced aspects of professionalism such as communication, respect, ethics, and accountability. The students' journal reflections revealed that they appreciated the different perspectives and roles of each profession in patient care, and that they learned how to communicate effectively and respectfully with other health care professionals to improve patient outcomes and safety. The students gained confidence and skills in working with other health care professionals to coordinate patient care and resolve conflicts. They also recognized the importance of system thinking and			Time constraints- The students only participated in one case-based scenario, which may not be enough to solidify their communication and collaboration skills.	not menioned	Co-designed	Not soley HCP?9
45	Waller	Interprofessional simulation in a student community clinic: insights from an educational framework and contact theory.	Advances in simulation	2019	Australia	explore the stakeholders' perceptions and experiences of training for, and conduct of, a simulated client-based activity to support the development of collaborative practice of students	Qualitative study	medicine+ nursing + physiotherapy+ exercise physiology + allied health assistance + occupational therapy + podiatry	Simulated participant	Name immediate obstetrical measures in the presence of postpartum hemorrhage and demonstrate these hands-on in the scenario.	simulated clients representing real scenarios from the local community health context	Feedback from simulated patient and discussion with faciliator after the scenario	holistic approach to client interviewing	improvements in students' communication and awareness of interprofessional collaboration. increased understanding of discipline differences and how to work together for better patient care.		Fustrations with technology. Duration of simultation being long 38 mins without feedback, no 'pause and discuss' techniques used.	safe space	co-designed interprofessional. deliberate purpose oV8:V16f performing a task together that was not discipline-specific but emphasised common collaborative competencies such as goal setting	
46	Van Wyk	Simulation as an educational strategy to deliver interprofessional educatio	AFRICAN JOURNAL OF HEALTH PROFESSIONS EDUCATION	2020	South Africa	aim of the study was to determine the opinions of the module leaders in the undergraduate programmes in the Faculty of Health Sciences, UFS, on using simulation as a learning strategy in IPE.	Quantitative study	dieticans + optometry + OT + Medicine + Nursing + Physiotherapy	Other	Team Communication:	structured interview with first author		ndicated that simulation sessions and the use of debriefing/reflection increased the students' understanding of a problem.	better clarification of roles among the different professions. simulation would be beneficial to their module(s) and enhance clinical training, and that small-group learning. could improve interprofessional teamwork, empathy and respect for other professions.		challenge of 'scheduling'. 'logistical and high cost issues' .	safe environement for students and patients		Not actual simulation- interview with module leads to look at views towards use of simulation in IPE
47	Aljahany	Simulation-Based Peer-Assisted Learning: Perceptions of Health Science Students	Advances in medical education and practice	2021	Saudi Arabia	this study evaluated the perceived advantages of simulation-based peer-assisted learning among health professions students and interns and their acceptance of this new concept of learning from a student-instructor	Mixed methodology	Medicine + Dentistry + Pharamcy + health + rehabilitation science	Other	Re-evaluate the situation according to the 10-seconds-for-10-minutes principle.	provided with a workshop about designing and implementing simulation activity. Students assigned to 4 interdisciplinary workgroups to write a simulation scenario	Not discussed	participants reported SB-PAL to be a new experience and beneficial for their learning	PAL provided good communication between the student tutor and their peers		complain of not sharing knowledge. academic schedule congestion. attendees were predominantly medical students who shifted the topics to focus more on medically related topics, and difficulty in extending and sharing information		no details provided on who facilitated sessions	Other simulation used- practical session on simulation scenario writing
48	Fonda	Interprofessional peer assisted learning: Paramedic students learning falls assessments from Occupational Therapy students	Journal of interprofessional care	2023	Australia	Pilot study assessing impact of interprofessional PAL	Mixed methodology	Paramedics + Ocupational Therapy	Simulated participant	Become confidently aware of one's role in the team (leadership, support coordination) during an emergency and act accordingly.	Peer-assisted learning (PAL). simulated case of a patient who had fallen.	provided direct feedback to the Paramedic students during a case simulation. No formal debrief described.	students reported improved self-perceived knowledge, skill, and confidence on of falls' assessments	high levels of engagement in simmultation self reported by students (HOWEVER higher levels reported by OT than paramedics)		ensuring engagement- differences between professions ideas about professional roles and hierarchy		lead by OT facilitator to paramedic students. No clarification re design of scenario	

49	Burnett	Preparing future health professionals via reflective pedagogy: a qualitative instrumental case study	Reflective Practice	2019	USA	simulation exposed students to the difficulties associated with poverty. Reflect upon ethical decision-making and their future role as a health professional. The primary purpose of the current study was to expose undergraduate public health education and health studies students with a hands-on, interprofessional, transformative learning experience by participating in a bi-annual poverty simulation on-campus and writing a reflection paper on their experience	Qualitative study	public health education majors + health studies majors	Simulated participant	Missouri's CAPS model. Students are appointed a particular role within a randomly assigned low-income family unit. The overarching goal of each family is to provide basic necessities during four 15-minute segments. Throughout the simulation, each family continuously interacts with various community agencies.	three-hour hands-on simulation	Students also engaged in a debriefing session during one class period	development of empathy skills, development of character in relation to professional identity, understanding of professional responsibilities	ne student observed, 'short-term and long-term outcomes are best achieved when everyone works together.'	incorporate ped agogical activiti es, such as simulations, to allow undergraduate students to foster interdisciplinar y team-building skills.			CAPS by the Missouri Community Action Network. Exact scenario design not discussed.
50	Delbridge	Working at a cultural interface: co-creating Aboriginal health curriculum for health professions	Higher Education Research and Development	2020	Australia	co-create a curriculum for undergraduate and postgraduate health professions programmes with Ab original expert partners with the intended learning outcome to develop students' cultural ca pability for professional practice	Mixed methodology	dietician + psychology + Nursing + Occupational Therapy	Simulated participant	Develop students cultral capacity and interprofessional attitude. prepares students to provide culturally safe healthcare for Aboriginal people in interprofessional healthcare settings	simulation was based on Tag Team Patient Safety Simulation (TTS). Impact of Aboriginal culture on health in inpatient rehabilitation.	Pendleton feedback model. Feedback on students interactions and TTS participation	significant pre-to-post improvements in students' self-rating of respect, communication and safety	significant pre-to-post improvements in students' self-rating of respect, communication and safety			TTS requires no specfic simulation environment or equipment	co-designed interprofessional
51	McCrea	Collaborative Interprofessional Health Science Student Led Realistic Mass Casualty Incident Simulation.	Healthcare	2022	USA	This study aimed to evaluate a cross section of student health care providers, both pre-hospital and hospital based, through a realistic interprofessional education (IPE) simulation training event to determine their working knowledge of the processes of the National Incident Command System (NICS).	Mixed methodology	public health + informatics + biomedical sciences + medicine + nursing	Simulated participant	develop understanding of emergency skills i.e. triage	simulation drill staged at a 15-acre, urban fire department academy utilizing real property designed to train emergency teams. bus of hurricane evacuees, some with special health care needs that washed off a bridge during an evacuation. The event lasted two hours	students were encouraged to reflect and share their experiences, what they had learned and what may have been unexpected. Students were asked what they felt went well and significant areas for improvement. The faculty concluded the debrief by highlighting how this simulation could be applied in a real-life setting	Clinical skills and reasoning development- including triage and basic first aid, detecting biological agents, and knowledge of procedure and administrative command structures. Most improvement on the dimension of assisting with Triage (START). students expressed they felt more capable to provide care		Students discussed an overall feeling of overwhelm during the discussion and debrief.			co-designed interprofessional
52	Fenzi	An Undergraduate Interprofessional Experience with Self-Learning Methodology in Simulation Environment (MAES©): A Qualitative Study	Nursing Reports	2022	Spain	impact that a Self-learning Methodology in Simulated Environments can have on Interprofessional Education within a Crisis Resource Management simulated scenario (MAES©)	Qualitative study	Medicine + Nursing	Full body manikin	better training for both technical and non-technical skills within Crisis Resource Management	Self-learning Methodology in Simulated Environments. Crisis Resource Management scenario. case was set in a pre-Surgical area. The patient was a young female with acute abdominal pain that required emergency surgery. She needed airway management, and it was during her airway isolation that a crisis situation arose	structured debriefing (5-min briefing, 10 min simulation, 30 min debriefing, and 15 min exposition phase). standardized debriefing following the MAES© standards took place and was guided by a facilitator. The students analyzed their strengths and weaknesses and implemented their knowledge (exposition phase).		students expressed could enable an opportunity for implementing teamwork and communication, with a better understanding of all the situations. sharing competences between different health professionals		students expressed initial difficulty when adapting to students from a different degree and with different education and careers		co-designed interprofessional
53	Kuehn	A Poverty Simulation's Impact on Nursing and Social Work Students' Attitudes Towards Poverty and Health	Creative Nursing	2020	USA	assess students 'attitudes about poverty and beliefs about the relationship between poverty and health	Mixed methodology	Nursing + Social work + Physical therapy + Doctor of nursing	Simulated participant	simulation allowed students to glimpse poverty and work with families over simulated 1 month period with different agencies	CAPS by the Missouri Community Action Network. simulation depicted 26 families, each student assigned to one family. X4 15 minute sessions	hour-long debrief after the simulation allowed students to discuss and reflect on their experience with the whole group	Study focuses on impact on learning related to thoughts/ beliefs in relation to poverty. No dicussion in results on IPE	Not discussed		Not discussed	Not discussed	CAPS by the Missouri Community Action Network. Exact scenario design not discussed
54	Baughn	Undergraduate Nursing Application of the Rapid Aspiration Screening Tool (RAS-3): An Interprofessional Swallow Screen Simulation	Clinical Simulation in Nursing	2023	USA	interdisciplinary simulation provided with an opportunity to participate in their respective professional roles when administering a swallow screening (RAS-3) to a standardized patient with suspected stroke	Mixed methodology	Nursing + Speech and language	Simulated participant	interprofessional compentency- swallow screen (Rapid Aspiration Screening [RAS-3].	standardized training protocol and simulation activity using standardized patients. pre briefing session RN and SLP students were randomly partnered with each other in pairs, with the former tasked to administer the RAS-3, while the latter rated the accuracy and completeness of RN administration of the RAS-3 using the RN competency sign-off sheet	simulation portion, students attended a group debriefing session with course instructors (C.B. and K.G.). These sessions were intended to discuss the students' experiences and perceptions regarding what they learned, benefits of the simulation, challenges they faced during the activity, and ways to improve interprofessional collaboration between RNs and SLPs.	improved competency scoes pre and post simulation activity. Standardised patiets helped prepare for clinical practice. benefits of the simulation on gaining practical clinical skills and experience	simulation provided them with an opportunity to engage in interdisciplinary interaction, which they seemed to recognize as valuable to their learning and future clinical practice. Interdisciplinary simulation promotes a low risk, high fidelity clinical scenario that models interprofessional collaboration and clinical reasoning in the preprofessional environment. The postsimulation debriefing also allowed students to engage in interdisciplinary communication about their professional roles, responsibilities, and experiences, and provide feedback to their student counterparts.		plans for continued interdisciplinary activities may be challenging considering conflicting program schedules, faculty obligations, and the size of student cohorts.		co-designed

55	Brashers	Measuring the impact of clinically relevant interprofessional education on undergraduate medical and nursing student competencies: A longitudinal mixed methods approach	Journal of interprofessional care	2016	USA	The purpose of this article is to describe a three-year project at the UVA to develop innovative clinically relevant undergraduate IPE experiences based on CCBPMs, integrate these experiences throughout the clinical/clerkship years of third-year medical and nursing students, and assess the impact of these experiences on students' achievement of IPE competencies.	Mixed methodology	Medicine + Nursing	Mixture	CCBPMs were used to create IPE simulation experiences for clinicians and tools that assessed specific collaborative behaviours	four IPE simulations were scheduled and integrated as required workshops during nursing clinical courses and medical clerkship rotations. Four simultaion situations were: difficult discussions, rapid response, paediatric illness and transitions in care. In the four scenerios: x3 used simulated participants and one used high fidelity simulator manikin (rapid response scenario). Outcome measures: team skills scale (TTS), Collaborative behaviours observational assessment tool (CBOAT), interprofessional teamwork objective strutured examination (ITOSCE).	1) Difficult discussion workshop: workshop ends with debriefing about lessons learned and further discussion of the CCBPM-specified behaviours. 2)Rapid response workshop: Faculty observe both phases of the simulation and provide immediate feedback as well as debriefings during which the CCBPM behaviours are reviewed. 3) paediatric illness: Each scenario is followed by a debriefing discussion. 4) transitional care: debrief not discussed	Self reported progression in competency skills	Comments in free text relating to interprofessional bridging of barriers. "I think we should always work with RN students because it's good for inter-professionalism and it more realistically simulates what will happen." workshop helped them to see the value of teams in providing safe and effective patient care	No identified difference in learning outcomes achieved between medical ndnursing students noted.	learning to work in teams can pose a threat to achievement of one's professional identity, it is important to integrate profession-specific skills with those interprofessional skills that should be gained by all professions. This integration can help learners contextualize their professional identity as a part of their team identity.	IPE must be dynamic and responsive to the complexities of different practice settings and the specific behaviours unique to collaborative teams in those settings. Learners value and retain more positive perceptions of IPE if the activities provide opportunities to interact with students in other professions to address clinically relevant problems.	co-designed. CCBPM approach used in this study provides a model and strategies for creating IPE activities that are based on clinical guidelines for specific practice areas and that integrate profession-specific behaviours with interprofessional behaviours
56	Brown	The use of trauma interprofessional simulated education (TIPSE) to enhance role awareness in the emergency department setting	Journal of interprofessional care	2016	UK Scotland	aim was to explore the views of radiography, nursing, and medical students regarding preparedness for trauma practice	Mixed methodology	Medicine + Nursing + Radiography	Full body manikin	main objectives were to increase IPSE and prepare students for professional practice in trauma.	In pairs or triads, radiography students were given a simulated scenario, using SimMan 3G Trauma (Laerdal), along with nursing and when available medical students. All scenarios involving "trauma radiography" were led by a qualified emergency department physician with specialists in both nursing and trauma radiography. All sessions were performed in situ within the resuscitation room of the hospital to enhance simulation fidelity.	Not discussed	increased preparedness to deal with trauma scenario.	Post-scenario felt significantly more prepared to undertake their role in the team and had better understanding of their and other professions' roles in trauma. Increased in students understanding of their own and others roles/ responsibilitie siwthin the team "uncertainty as to others responsibilities within the team".	did not appear to be any major differences when analysing individual professional groups	Not discussed		co-designed
57	Carpenter	Interprofessional Collaborative Practice: Use of Simulated Clinical Experiences in Medical Education	The Journal of the American Osteopathic Association	2018	USA	This study was conducted to determine osteopathic medical students' perceived value and effectiveness of simulated clinical experiences (SCEs) in cultivating interprofessional competencies.	Mixed methodology	Medicine + Nursing	Full body manikin	experiences were designed to simulate the first days of clinical rotation as residents.	performing a history and physical examination on a high-fidelity human patient simulator. The medical case scenarios included acute myocardial infarction, metastatic lung cancer, long-bone trauma, and other common disease states. Medical students were assigned to work with a nursing student in completing the assigned tasks. The students were asked to report their findings to the attending physician in a standard SOAP (subjective, objective, assessment, and plan) fashion. The students then discussed the status of the patient with the family, portrayed by actors, who followed scripts to present difficult or stressful scenarios.	After completing the SCEs, they participate in a comprehensive debriefing with medical and nursing faculty, the patient's nurse, and the actors portraying family members. Students were debriefed while watching a video-recorded playback of their personal simulation experience. Nursing staff and medical instructors provided constructive feedback and prompted students to personally reflect on the experience.	Questionnaire highest agreement rating with- "I feel better prepared to communicate with family members" and "I was challenged in my thinking and decision-making skills". They bore the responsibility of communicating interpersonally during the experience, including delivering bad news to families, a skill that is technically and emotionally challenging.	we demonstrated the importance of collaboration among different health care professionals to establish mutual respect. Students were able to take ownership of their roles as future medical providers to adequately serve the needs of their patients.		The study was administered during a variety of rigorous academic events, eg, final examinations and board preparation periods. Thus, some students may have felt less prepared than others, owing to distracting academic obligations.	enables students to overcome any fear of interacting with patients, nurses, and families by providing a safe environment in which to make errors	no discussion surrounding case design
58	Cooke	Tracing the prescription journey: a qualitative evaluation of an interprofessional simulation-based learning activity	Advances in simulation	2017	UK (NI)	innovative IPE activity was developed for medical and pharmacy undergraduate students that aimed to develop a greater understanding of their roles and duties in community prescribing and dispensing. This study set out to evaluate the impact of such a SBE activity on students' attitudes towards collaborative practice in prescribing and dispensing medication in the community. Primary aim of this project was to qualitatively evaluate the impact of this SBE activity	Qualitative study	Medicine + Nursing	Simulated participant	IPE team clinically assessing, diagnosing, writing prescriptions, dispensing medication(s) and counselling a simulated patient (in a simulated practice and pharmacy setting)	small mixed-disciplinary groups, they were asked to consult with a 'patient' in a simulated GP office. The simulated patients described common general practice presentations (e.g. back pain, sore throat, primary cardiovascular risk assessment and request for emergency contraception). During this 'consultation', students were provided with a medical chart relating to the patient, access to a drug formulary and other diagnostic equipment. Typically, during this 'consultation', the medical student would lead, with the pharmacy student actively observing. student small groups were tasked to consider a working diagnosis for the patient. They then collaborated on writing a mock drug prescription and detailing a management plan. Following this, they engaged with the simulated patient again to explain their working diagnosis and negotiate their proposed management plan. After this was complete, the simulated patient attended the 'simulated	small groups were debriefed by faculty staff from the medical and pharmacy schools and the simulated patients. In these debrief sessions, feedback was actively encouraged between the two different groups of healthcare students in an attempt to reinforce interprofessional values and understanding. Whilst the simulation activity was not video-recorded, the faculty used their observations and field notes to guide the debrief session. The session concluded with a review of the evidence base on how best to manage their patients 'conditions'.	simulated environment contributing to their understanding and learning of the social and interpersonal dimensions of prescribing and dispensing. simulated patients provided them with a more realistic view of the problems. foster their mentorship abilities	IPE simulation activity: 1)creating a broader learning experience (typically IPE tended to be more theoretically orientated- classroom based learning).opportunity to apply their theory knowledge into 'practice' collectively and safely. 2)patient-centred practice: a shared understanding. IPE activity strengthened the trust and reliance they had in each other's knowledge and skills- prescription writing and counselling. 3)professional skills: explored and shared; and professional roles: a journey of discovery, respect and stereotypes. Understand how professional conflicts arise- discussion specifically around use of guidelines (medics flexibility, pharmacitist- strict adherence).		Perceived hierarchy and sterotypes: Pharmacy students commented that medical students can often seem intimidating in their knowledge at times, a notion which has been recorded before. pre-formed misconceptions among students could affect future attempts of collaborative practice.	structured activity gave students the opportunity to learn from each other, interestingly, pharmacy and medical students expressed simply being comfortable with each other and talking with their interprofessional peers further improved co-professional relationships. Some have noted the idea that non-classroom-based, informal networking parallel to IPE interventions should not be over-looked in the development of IPE curricula	co-designed
59	Cropp	Interprofessional pharmacokinetics simulation: Pharmacy and nursing students' perceptions	Simulation in Pharmacy Education and Beyond	2018	USA	This study describes student perceptions of an interprofessional pharmacokinetics simulation. Student perceptions of the session content, interprofessional collaboration, and the use of simulation were sought.	Mixed methodology	Nursing + Pharmacy	Full body manikin	Objectives for the pharmacy students were to: (1) provide optimal drug dosing using population and patient-specific pharmacokinetic parameters; and (2) communicate effectively in the care of a patient. Objectives for nursing students were to: (3) apply principles of leadership through interprofessional collaboration, and (4) promote medication safety through interprofessional collaboration with pharmacy in patient-centered care.	medium-fidelity manikins. Each case represented a pharmacokinetic dosing consult (vancomycin, tobramycin, phenytoin, theophylline, or lidocaine). Nursing students completed head-to-toe assessment and pharmacy students gathered necessary information and calculated empiric and adjusted doses. Students communicated using SBAR (Situation, Background, Assessment, and Recommendation). Total five cases.	debrief sessions included all students sharing their perceptions of the collaboration, demonstrating the collegiality that had developed among them.	Many students stated that they enjoyed the simulation experience as it allowed hands-on practice with real-life scenarios to encourage collaboration and critical thinking.	Interprofessional collaboration emerged as the overarching theme from the IPE event. Five major areas emerged from the overall thematic analysis for the research: Interprofessional collaboration; interprofessional communication; values and ethics; roles and responsibilities; and teams and teamwork. Other themes that arose from the data included: Safety; advocacy; appreciation for colleagues; patient-centered care; knowledge for practice; applying knowledge and evidence; professionalism; and improvements for the future. Students expressed the need for greater communication with team members in the future, as well as increasing confidence in interacting with the other profession.	Although most students voiced preference for learning in the simulation environment, some students expressed the need for classroom lectures to set the foundational knowledge	professional stereotypes and perceptions of other disiciplines skills and knowledge base. emphasize the professionalism of collaboration and importance of clear communication of within-profession jargon to the other profession in the development of collaborative IPE experiences.		co-designed. The cases were developed in conjunction with the instructor of record for the pharmacokinetics course, who also co-led the lab activity.

60	Data	Feasibility of an Interprofessional, Simulation-Based Curriculum to Improve Teamwork Skills, Clinical Skills, and Knowledge of Undergraduate Medical and Nursing Students in Uganda: A Cohort Study	Simulation in healthcare : journal of the Society for Simulation in Healthcare	2021	Africa (Uganda)	aimed to evaluate the feasibility of implementing an undergraduate IP, simulation-based curriculum and to describe the possible impact of this curriculum on teamwork skills, CSs, and knowledge as measured at baseline, 1 month, and 10 months among a cohort of medical and nursing students in Uganda. We also aimed to determine whether teamwork skills were transferable across different clinical scenarios by the end of the curriculum.	Prospective Review	Medicine + Nursing	Full body manikin	development of team work, clinical skills and knowledge	Simulation sessions were conducted in the university medical simulation center. Simulation rooms were equipped with audiovisual recording equipment to capture video recordings of all simulations. We used the Laerdal NeoNatalie (for the neonatal sepsis scenario), Laerdal MamaNatalie (for postpartum hemorrhage), and the Laerdal Resusci-Anne (for adult sepsis and postpartum bleeding) manikins in the study. The medical equipment provided was setup to reflect availability in the real clinical environment. Scenarios were time limited to 20 minutes. 3-minute prebriefing video. video was followed by an opportunity for participants to orient themselves to the environment and manikin functionality.	facilitated debriefing was conducted by a trained facilitator after each simulation scenario. We used the Promoting Excellence and Reflective Learning in Simulation–blended method debriefing framework to facilitate debriefings. All debriefings were scripted to include key medical and teamwork discussion points. The purpose of these debriefing scripts was to ensure that in addition to learner generated topics, all teams were exposed to key learning objectives within the 20-minute debriefing period.	Variation between clinical cases with some showing an increase in clinical knsowlege whilst other not. Clinical skills showed no significant improvement at 10 months compared with baseline; however, knowledge of rotation-specific content was acquired and retained for all 4 content areas.	Improvement in team work skills. This IP simulation curriculum seems to be associated with acquisition and retention of teamwork skills that are transferable to a different clinical context.	IP simulation curriculum for undergraduate students is feasible in a low resource setting		stakeholder engagement. institutional investment in simulation infrastructure. Sustainability can be further enhanced if faculty and students have dedicated time allocated to medical simulation. safe, low-risk environment for healthcare trainees and providers to practice effective teamwork. everal variables contributed to transferability of teamwork skills across contexts in our study. With the design of our study, participants were in the same groups throughout the year, thus providing them opportunity to refine their performance as a team over the year. The opportunity to apply teamwork principles in different contexts, coupled with facilitator feedback during debriefing, likely helped reinforce positive behaviors. Lastly, regular	co-designed. Students, teaching faculty, and university administrators were engaged in planning and simulation perception studies that helped in understanding anticipated simulation implementation challenges and opportunities specific to our setting that were vital in curriculum design and implementation.	
61	Anderson	Taking a closer look at undergraduate acute care interprofessional simulations: lessons learnt	Journal of interprofessional care	2020	UK	paper reports on the evaluation of the UIPSims completed by students at the end of their training. The findings were intended to feed back into the IPE curriculum theme to illuminate whether students were ready for interprofessional collaborative practice and also to highlight how to better prepare and support students in an interprofessional simulation.	Mixed methodology	Medicine + Nursing + Pharmacy + operating department practitioner	Full body manikin	illuminate whether students were ready for interprofessional collaborative practice	simulation the role of the patient was fulfilled by a high-fidelity manikin. simulation center which had been modelled on a modern ward, consisting of 4 bay areas, each with 4 beds. For the completion of these UIPSims, students were assigned to small interprofessional groups which rotated around the 4 ward bays. In each bay one situation was staged; students either watched, seated away from the activity as observers, or stepped up to be participants. Every student was an active participant in at least one bay. The simulations in the four ward bays comprised, i) a patient admission, ii) a patient transfer to surgery, ii) a collapsed patient on the floor and iv) a patient requiring a written prescription. facilitator introduced the simulation to the students in a briefing and led a post scenario student group debrief, allowing students to participate in problem-solving and consider new strategies for moving forward. Each simulation lasted approximately 30 minutes	Debriefing followed accepted models, allowing students time to relax, share immediate emotions and provide descriptions of what happened, including what went well and what could have been improved. The facilitator guided learning implications and involved participants and the observing students	Themes included the importance of learning in a real clinical setting. others spoke of about becoming more self-aware concerning human factors and patient safety	1) team work and leadership- Team working and how people interact was a prominent theme. Leadership and followership was haphazard; sometimes the leader became so dominant that other team members were marginalized, occasionally to the point where they contributed little. 2) interprofessional education- Intra-team communication tended to be erratic, with prime movers sometimes not vocalizing their reasoning, actions and conclusions. Participants seemed unaware of the use of structured communication tools such as SBAR. Largely negatively focused outcomes from study regarding aditional factors such as situational awareness.	advocate that students should not come together for UIPSims until they have been given the knowledge, skills and behaviors appropriate to the 'craft' of teamworking and have spent time in simulation units both uniprofessionally and interprofessionally so they are confident and familiar with the environment.	some students, such as therapists and pharmacy students, are less likely to have access to these acute clinical simulation settings when compared to nurses and medical students. As a result, these students are less comfortable in the simulated environment and less prepared to engage in the learning. observer noted that on several occasions the student teams were large. This impacted on the clinical space for the simulation. In addition, the numbers of students and variations in professional mix sometimes made it difficult to allocate students to balanced interprofessional teams. the behavior of some students indicated they had little prior knowledge of the technical capacities of the high-fidelity manikins. Lacking this knowledge, students were sometimes left wondering what	The relaxed friendly and fun learning environment helped them work together. pose questions for curriculum planners because it could be argued that leaving such UIPSims to later in a curriculum leaves little time for students to improve- needs introduced from early in curriculum. advocate for uni-professional before interprofessional preparation. we argue that the emphasis in UIPSims must move toward briefing rather than focus on de-brief.		
62	Feltham	Student midwives and paramedic students' experiences of shared learning in pre-hospital childbirth	Nurse Education Today	2016	UK	aim to explore the experiences of midwifery and paramedic students undertaking interprofessional learning	Qualitative study	Midwifery + Paramedic	Full body manikin	investigating the experiences of both midwifery and paramedic students' perceptions of shared learning in the context of pre-hospital childbirth	high-fidelity pre-hospital obstetric scenarios was undertaken (no exact details on mannikin type provided), where students collaborated in small teams performing their respective roles. 6 hour workshop- ice breakers and discussion of each professions roles and respondsibilities in AM session, simulation in PM session. No discussion of cases used for simultaion.	No debriefing discused. midwives commented however that they felt there was no proper closure to the day following the simulation based learning session, and would have valued all meeting up together at the end of the day for a collective debriefing.	placed value un 'unplanned learning'. Several of the first year midwifery students felt however that they lacked the knowledge of their paramedic counterparts and vocalised how self-conscious this made them which impacted on their enjoyment of the day.	Four main themes were identified around the understanding of each other's roles and responsibilities, the value of interprofessional learning, organisation and future learning. he day enhanced the students' understanding of each other's roles and all the students felt this was reciprocated. Having time to engage on an informal level enabled them to gain a valuable insight into the roles and responsibilities of paramedics and all students felt a mutual respect was fostered. understanding of the skills and limitations of each other's role.	negative association with simulation based learning with some midwifery students feeling uncomfortable engaging in 'role play'. More negative feedback from junior midwifery students- suggest that senior students may engage more readily with simulation based learning because they have a better understanding of their roles and responsibilities and may feel	Timing of when to introduce IPE into circculum too early- students lack knwoeldge to fully engage in the activity and lack groudning in their professional roles.	use of 'icebreaker' exercise pre simulation.	no discussion surrounding case design	
63	Flossel	Using simulation mannequins and actors in training for external post-mortem examinations - experiences from use in medical students and police officers	Journal of forensic and legal medicine	2021	Germany	the goal of this study was to present the experiences from using simulation mannequins and actors for external post-mortem examination training in medical students and police officers.	Survey	Medicine + Police	Full body manikin	Medics: learning objectives for the medical studentsinclude: empathic interaction with the relatives; conducting a complete external post-mortem examination in scene of death; and determining the cause and the manner of death. Police: ost important learning objectives are the correct evaluation of criminality of the situation and circumstances of the discovery, such as the necessary examination of the conditions at death, appropriate initial consultation with the relatives, following proper forensic procedures and documentation at the location where the cadaver is discovered, as well as ensuring effective cooperation with the medical professional during external post-mortem examination.	6 different stations and carried out in small groups of 3–5 people. After instructions are given by a trained student tutor, including a brief review of the theoretical basics, the students receive the description of the scenario and their working task. On entering the training room, they will be faced with a typical domestic situation. They are greeted by an actress who plays the role of a relative of the deceased. A lifelike and fully dressed mannequin represents the deceased. Police: After an initial interview with the relative, follows a visit to the discovery location, forensic services like photographic documentation are carried out, and an examination of the simulation mannequin (playing the deceased) is carried out in collaboration with a doctor (student tutor).	No formal debrief discussed. At the end of the exercise, the students received an evaluation and structured feedback from the tutor.	students commented on high practical relevance of the scenaio. Limitation- "external post-mortem examination on mannequin is unrealistic and not effective"	Students not asked specific questions relating to IPE this limited data collected in this area of interest. Medics and police officers also had distinct learning outcomes		separate adapted scenario for medics and police, separate learning outcomes- poor integration of the simulation between professions	ensure very good cooperation structures with the respective medical learning centre and external cooperation partners		Limited study- limited direct interprofessional working

64	Gorantla	Introduction of an undergraduate interprofessional simulation based skills training program in obstetrics and gynaecology in India.	Advances in simulation	2019	India	aims to evaluate the effectiveness of an education program using simulation in teaching core clinical skills to medical and midwifery students by assessing students' perceptions of the program. Additionally, by having medical and midwifery students learn together, it explores the benefits of early interprofessional training in obstetrics and gynaecology.	Mixed methodology	Medicine + Midwifery	Task manikin	learning objectives were relevant to both professional groups, which included safe handling of instruments, ensuring patient safety and comfort during clinical examination, conducting clinical procedures of low complexity as a team of medical and midwifery students and identification of complications.	3-h clinical skills workshop, which was complemented by a blend of online-lectures, pre-reading material and videos provided to the participants beforehand. skills station circuit was formed, where groups of 6–8 students practiced for an hour at each station, which was led by a facilitator. The facilitator team comprised of local midwifery and medical staff with extensive experience in the fields of obstetrics and gynaecology and were also involved in teaching medical and midwifery students. stations consisted of the following skills training: 1.Speculum, bimanual examination and performing a cervical screen test, 2. Vaginal examination and assessment in labour, 3.Conducting a normal vaginal birth with estimation of blood loss. Zoe Gynaecologic simulator and PROMPT flex birthing simulator.	No formal debrief discussed. Individual and group feedback was given to the students at each skill-station.	main theme that appeared throughout the post-workshop survey was that the workshop provided "hands-on" learning	six themes identified from the text data were getting hands-on practice; learning in the simulation without clinical time constraints (in clinical environment can feel hurried and burdensome); retaining the ability to make mistakes; bridging theory to practice; valuing interprofessional experience (coordinate and share their ideas and knowledge in a mutually beneficial manner, expressed greater trust and understanding in one another) ; and ensuring equal learning opportunities for all participating professional groups	midwifery students were more aware of interprofession al education than their medical student counterparts. midwifery students felt that the medical students were more dominant during the IPE and deprived them of the chance to participate equally in the procedures.	quality of opportunity offered to both sets of students and dominance of one group over another may occur in an IPE setting. While both groups of students appeared to be immersed in the activity, the results suggested an underlying perception of contest about learning opportunities. These perceptions may potentially weaken the development of collaborations and role-sharing, that IPE activities intend to foster. ow- and middle-income countries like India, a strong hierarchical system is still prominent in the healthcare sector.	Developing a supportive environment of psychological safety between the two interprofessional teams, where individuals feel comfortable participating in discussion and raising opinions without fear of being judged, criticised or ridiculed, will be essential	he program content was matched to both the medical and midwifery curricula and was designed and approved by both curriculum leaders	
65	Gordon	Partnering for Patti: Shaping future healthcare teams through simulation-enhanced interprofessional education	Canadian Journal of Respiratory Therapy	2017	Canada	primary objective of this research was to determine if participants' understanding of CIHC IPE domains improved, and if perceptions of their own and the other profession were reframed as a result of this innovation. Canadian Interprofessional Health Collaborative (CIHC) has established a national competency framework of integrative competency domains focused on fostering core skills, attitudes, and values in an effort to evolve interprofessional	Mixed methodology	Nursing + Respiratory therapy	Full body manikin	overarching goal of this learning experience was to enhance student knowledge of the six CIHC IPE domains with a more focused goal of sensitizing learners to IPC.	medium-fidelity sim-IPE to depict a client who, during a shift assessment, presented with an acute change in respiratory status. event began with a prebriefing workshop 10 mins. Each group received a separate discipline-specific shift report for the client. Student nurses then conducted a routine assessment, in which an acute change in the client's status was noted. This change prompted student nurses and student respiratory therapists to collaborate to effectively meet the client's needs. Participants were assigned either hands-on or active observer roles. Simulation 20 mins.	30 min debriefing. Immediately following the simulation, students participated in a structured debriefing session. The advocacy-inquiry model guided the facilitated debrief, with a focus on the central CIHC IPE domains as the IPE learning objectives; to this end, educators consistently foregrounded IPC as the salient skill set, rather than technical discipline-specific skills. Lastly, students completed a post-simulation structured reflection assignment (2 hrs), following Johns' model , whereby participants critically examined and reflected on their perceptions and experiences as they related to the events	significant improvement in 'understand the capabilities of your profession' and 'fully utilize the capabilities of your profession'	Positive response- "communicating within and outside their discipline," and "collaborating with students from other healthcare disciplines." Role clarity was noted recurrently. improved understanding of CIHC IPE domains; content analysis suggests communication, collaboration, and role clarity were key elements of learning.	one group of students had undertaken the pilot of this study. Feedback from this group in this study more negative (?related to repeat participation- key learning took place in pilot?)	students often strayed from the main purpose of this sim-IPE event. This occurred during times where case acuity or participants' abilities to solve physical or task related problems in response to the client's condition became the priority both within the simulation and during the debrief rather than collaboration and effective team communication. widely recognized that some health professions (such as nursing) have substantially larger class sizes; thus, producing groups that have an even distribution of professions is an ongoing challenge in IPE, largely due to time and resource constraints.	the criticality in making sure the simulation scenario aligned with both specified competencies and student ability. importance of foregrounding learning objectives at the outset.	co-designed	
66	Greig	'The most useful exercise of medical school': simulated death can be successfully incorporated into undergraduate simulation.	BMJ simulation & technology enhanced learning	2018	UK (Oxford)	assess the role simulation has to play in preparing students for these emotionally challenging events such as death of a patient	Mixed methodology	Medicine + Nursing	Full body manikin	Scenarios are graded in their complexity. The first patient of each session requires only appropriate assessment and investigations. The second scenario simulates a critically ill patient with a non-fatal condition, and the final scenario ends with the patient's death despite all appropriate management.	Sessions comprise three 30-min scenarios over a half-day. A METI HPS manikin simulates the patient, an actor plays a family member, and an extended faculty of junior, middle-grade and senior doctors, and a nursing sister is provided. maximally realistic scenarios. students experience events that simulation has traditionally avoided, including patient death. Scenarios are managed by teams of two second-year nursing students and three final-year medical students. After a brief handover, nursing students begin treating the patient, and bleep the medical team to attend at an appropriate point. Students are never asked to work outside the role of a newly qualified FY1/staff nurse and are free to call on senior support. Scenarios are performed in real-time, and students are required to cannulate the manikin, prescribe all drugs and draw and label samples correctly. Students are provided with a simulated drug cupboard, including a lockable controlled-	Each scenario is followed by a 45-min debrief. Debriefing following this event is carefully conducted, exploring student reactions and emphasising that death can occur despite correct treatment. Indeed, it is highlighted in the debrief that the manikin died despite what the team did, not because of it. This appears to be a novel thought for most students.	surprised by how difficult the performance of practical tasks could be under pressure, and how much this impacted on non-technical skills such as situational awareness. frustration experienced when failure to accomplish tasks impacted team management.	developed understanding of own professional role and that of other team members. Developed team working and collaboration.	no feedback directly mentioned the death of the patient—this event does not appear to have registered with the students as either unusual or inappropriate. situation performed in real time- gave students an idea of how long these can take in practice.	not discussed	not discussed	not discussed	Limited study- focus more so on simulation of a patiet death than IPE
67	Hardisty	Simulating complexity: providing undergraduate students with exposure in early clinical training to the multidisciplinary management of frail older people	BMJ simulation & technology enhanced learning	2019	UK	aims to translate the clinical experience of the multidisciplinary frailty team into a classroom-based teaching session for undergraduate healthcare students	Pilot study	Medicine + pharmacy	Simulated participant	objectives of the project were the following: To simulate the complexities of the management of frail older people in the classroom. To assess the feasibility of delivering teaching and learning focusing on the care of older people and management of their medications in the early stages of clinical training. To assess the attitudes and acceptance of students towards interprofessional education and low-fidelity simulation as pedagogical methods for delivering teaching and learning around the care of older people. To provide a preliminary indication of the knowledge and skills gained by students through this initiative.	Two teachng cases developed. Teaching resources were produced, including simulated patient notes and other documentation such as NEWS charts and blood test results. Bags of medication (prepared to appear as if they had been brought in from a patient's home) were provided to support medicine reconciliation tasks. Students were given relevant guidelines and support materials including the Aging Brain Score.5 Further attempts were made to simulate the complexities of caring for a frail older person and the sensory and physical impairments that can affect activities of daily living. A low-fidelity old age suit was purchased and used to demonstrate the effects of visual, hearing and mobility impairments on medication administration. Students were given tasks including reviewing the patient's history, examination and investigations to create a problem list, performing medicine reconciliation, reviewing and amending medications and	Facilitators gave feedback on each of the tasks and provided their real-world experiences of working in the frailty team or within other contexts with older people.	Knowledge acquisition, Prescribing skills, Professional and reflective skills	Increased understanding of the multidisciplinary team		logistical challenges including timetabling to offer this opportunity to larger numbers of pharmacy and medical students	the relevance of the cases to both professional groups and the multidisciplinary facilitation	not discussed	Pilot study- limited detail within article

68	Jakobsen	Examining participant perceptions of an interprofessional simulation-based trauma team training for medical and nursing students	Journal of interprofessional care	2018	Norway (Oslo)	We developed a short simulation-based course for final year medical, nursing and nursing anaesthesia students, using scenarios from emergency medicine. The aim of this paper is to describe the adaptation of an interprofessional simulation course in an undergraduate setting and to report participants' experiences with the course and students' learning outcomes. aim of this paper is to describe the adaptation and implementation of the BEST (Better and Systematic Team	Mixed methodology	Medicine + Nursing	Full body manikin	one-day team training course for medical students, nursing students and students in nursing anaesthesia, in which high-fidelity simulation was used to train team skills in an emergency medicine setting	. Each team consisted of 4 medical students, 2–3 nursing students and 0–2 students in nursing anaesthesia. Each team performed four video-recorded simulations in real time without interruptions. four 60 minutes sessions consisting of simulation (15–20 minutes) immediately followed by debriefing (40–45 minutes), alternating with four interactive lecture sessions of 30 minutes including an evaluation session. All lectures were focused on the interprofessional aspects of the day, including roles in the team, communication between team members, leadership and tools for efficient teamwork.	45 minute debrief lead by faciliators immediately after the simulation. facilitators led a structured debriefing session, using the video recording of the team performance as a resource for reflection in the team, focusing on communication, leadership and intra- and interprofessional collaboration. All team members were invited to contribute, and members with less "formal" influence in the team were asked to comment first, e.g. nursing students commenting before nursing anaesthesia students, who spoke before medical students. The team leader invariably spoke last. At the end of each	Themes: Self-insight and stress management. emotional activation by simulation experience- both positive and negative expressed. Our finding of both negative and positive reactions highlights this paradox that must be taken into account when deciding how much stress to put in to a simulation scenario. value of communication techniques such as "closed-loop" and doing situational summaries with loud and clear messages.	Themes: understanding of the leadership role, insight into teamwork, and skills in team communication. a culture for mutual respect and interprofessional cooperation. students strongly agreed that briefings, summaries and closed-loop communication were important, and they reported having gained insights about communication, teamwork and leadership.	Medical students' scores were significantly higher on questions regarding leadership.medical students significantly agreed more than the other professions to be better suited to lead future teams, not unexpectedly as they were assigned the team leader role during the simulations. didactic lecture on debriefing and feedback was valued higher by nursing		a strength in general, that all students acted in their "native" role and were not assigned a different role or profession. Sufficient resources are crucial for simulation-based courses.	engagement of enthusiastic faculty from all professional groups involved was crucial, as they were able to influence how to make the course benefit their students during course development. Timing of the sim course in the curriculum- aligning with other modules being taught on leadership and teamwork. Trained facilitators from different disciplines- debrief trained, course on non technical skills in simulation.
69	Kleib	Interprofessional simulation to promote teamwork and communication between nursing and respiratory therapy students: A mixed-method research study	Nurse education today	2021	Canada	Aim to develop and evaluate an interprofessional simulation educational activity to promote teamwork and communication between respiratory therapy and nursing students	Mixed methodology	Nursing + Respiratory therapy		attending to what both disciplines would typically be exposed to in the clinical areas when caring for patients with these health conditions according to their professional scopes of practice	8 simulation scenarios, 15 minutes per scenario. Details regarding nature of simulation and cases used not dicsussed in article	10 minute debriefing after each scenario. final one-group debriefing session was held at the end of the day to facilitate reflection on IP simulation learning, followed by completion of a satisfaction survey, and focus group interviews with students and observes to share IP simulation experiences.		Pre test students most comfortable wit teamwork and communication, eats comfortable with role clarification, collaberative leadership, conflict resolution. Students strongly-agreed the training provided valuable team skills training (96%), the opportunity to communicate with students from other professions (100%) and a realistic experience of challenges faced when working in IP teams. agreed the simulation experience enthused them to learn more about IP (96%), helped them appreciate the importance of collaborative practice for patient safety (100%), enhanced understanding of own and roles of other members of the health team (92%), and they felt better prepared to participate in an IP team. Student-participants noted how the simulation	students recommended creating opportunities for students to learn about each other"s roles in informal ways	Student needs to feel comfortable within clinical skills element in IPE simulation as lack of familiarity can lead to increaed anexity.	(1) balancing complexity of learning scenarios with level of clinical skills of learners, (2) providing adequate orientation to the learning environment and scenarios in advance, (3) creating informal pathways to relationship and team building, and opportunities for students to educate one another about their roles and scopes of practice prior to IP simulation, (4) and increasing the duration of simulation learning, time for debriefing, and using less prescriptive and structured learning scenarios.	jointly developed by nursing and RT educators
70	Lee	Nursing and medical students' perceptions of an interprofessional simulation-based education: a qualitative descriptive study	Korean journal of medical education	2020	Korea	study aimed to describe the development and implementation of IPSE for undergraduate nursing and medical students and explore their perceptions of the impact of the IPSE and their changes in attitudes toward each other's health professions after participating in the IPSE.	Qualitative study	Medicine + Nursing	Full body manikin	learning objectives for their IPSE module that focused on interprofessional collaborative practice in clinical scenarios regarding deteriorating patients with acute myocardial infarction, and febrile seizure and postpartum bleeding after vaginal delivery. We made module learning objectives based on three domains of learning: knowledge, skills, and attitudes, considering the levels of fourth-year nursing students and third-year medical students.	2-day IPSE program that consisted of ice-breaking and patient safety activities, and 4-hour three interprofessional team-based high-fidelity simulation education sessions. IPSE program specifically designed for undergraduate nursing and medical students consisted of introduction, ice-breaking and patient safety activities, three IPSE modules, and reflection sessions. We developed the scenarios consisted of chest pain management in adult men with acute myocardial infarction, febrile seizure management in child, and postpartum bleeding management after vaginal delivery. One student group consisted of two nursing students and two medical students. IPSE program began with the facilitators using introduction and ice-breaking activities to build a sense of community and shared purpose.each student group was led by one nursing facilitator and one medicine facilitator. All participants in the groups took role-players and	Debrief after each scenario and at the end of the IPSE program, all participants gathered together in one place and had the time for a more thorough debriefing and reflection about their IPSE experience	IPSE experience helped them understand their own and medical professions' roles and responsibilities in providing health. alued the simulation as it provided the opportunity for hands-on practice in a safe, realistic environment.	Three themes emerged: "positive experience" with understanding roles and responsibilities and learning by doing in simulation environments being reported. In the second theme, "positive learning outcomes" participants reported enhancing collaboration and confidence in communication skills. The final theme "benefits to patients of interprofessional collaborative practice" included high quality of care and patient safety. After their IPSE experience, both nursing and medical students viewed each other as colleagues. Before the IPSE experience, medical students perceived the nurse as nightingale, bullying culture called as "taeum" in Korean, syringe, three shifts, and assistants. Their attitudes toward nursing profession were changed to nursing professionals, colleagues,		needing adequate rooms and facilities to accommodate a large number. Tend to be one off exercise and not completely integrated throughout a cirrculum.		Collaborative. We then reviewed, discussed, and refined these scenarios within each module team until agreement was reached. examined nursing and medical participants' simulation learning experience to develop IPSE program. Carefully considered each students group prior experiences of simulation in design process.
71	Leithead	Examining interprofessional learning perceptions among students in a simulation-based operating room team training experience	Journal of interprofessional care	2019	USA	to determine the extent of the impact of high fidelity SBT OR team training of interprofessional students for each profession. We hypothesised that such team training would have the same impact on all participants.	Mixed methodology	Medicine + Nursing	Mixture	High-fidelity simulation (HFS) is another training modality that has been used effectively to improve OR team function and attitudes	virtual OR, full-scale computer-operated human patient simulator mannequin and an inanimate torso module. Sessions lasted approximately 2 h and required a minimum of 3 instructors to be present. The instructors' roles involved operating the human patient simulator (HPS) and facilitating debriefing sessions. The same two standardised and authentic scenarios were used for each training session: life-threatening intra-abdominal haemorrhage from a stab wound, and local anaesthetic toxicity from a regional arm block. For a scenario, each OR team consisted of 2 medical students, 2 undergraduate nursing students, and 2 nurse anaesthesia students. Medical students assumed the roles of the surgeon and first assistant; undergraduate nursing students participated as circulating and scrub nurses; and nurse anaesthesia students served as primary and secondary anaesthetists. Students from each group switched roles after	All students participated in the immediate after action debriefing, which followed each scenario. The debriefings consisted of discussing the following team-based competencies: shared mental model, role clarity, situation awareness, cross-monitoring, open communication, resource management, flattened hierarchy, anticipatory response, and mental rehearsal. Debriefing technique followed evidence-based recommendations for effective debriefing in surgery. The session concluded in a final student reflection focused on strategies for incorporation of learned experience into clinical practice.	the degree to which each professional student group benefited from such SBT has remained unknown. current study sheds light on several important points related to this topic. First, HFS interprofessional OR team training has a modest overall impact on students' attitudes toward interprofessional collaboration and learning. Medical students and nurse anaesthesia students seem to benefit the most in this regard.	improves both team-based self-efficacy scores and observer-rated team-based behaviours and overall teamwork over the course of a training session. Anecdotal feedback by participants in this HFS training confirmed the value that students saw in bringing together the professional groups in the OR to learn from, about, and with one another. strong overall impact in terms of improvements in students' team-based attitudes, especially medical students and undergraduate nursing students. These two groups had positive mean changes in the 0.8 range in the IPT scale from pre- to post-session, and the difference in scores between professions was statistically significant. Nurse anaesthesia students did not have statistically significant mean changes in the IPT scale (hypothesised in this study to	Targeting students interested in careers involving work in the OR has the added benefit of fostering team trust and potentially negating the detrimental influences of the hidden curriculum	medical students typically outnumbered the other professions		collaborative

72	Lim	Dosage Form Modification, a Simulation Activity between Nursing and Pharmacy Students	Pharmacy	2022	Australia	aim of this exploratory pilot study was to evaluate student perceptions of a simulation activity involving undergraduate nursing and pharmacy students. The key question was "how do nursing and pharmacy students respond in an immersive collaborative simulation activity which involves medication dosage form modification?	Mixed methodology	Nursing + Pharmacy	Other	Simulation exercises were designed with face validity to replicate authentic situations through which student-centered interactive problem solving occurred without risks to patients.	Unclear if patient was simulated or simply worked through a case in a simulated clinical environement. simulated exercise where patients required medications for which there were barriers to administration. Fourteen pharmacy students were also present in the simulated health environment, observing the work of the nursing students and being available to provide advice regarding medication administration to the nursing students. clinical scenarios based in a medical/surgical ward. Students worked through four cases in the two-hour session in an immersive simulated hospital setting. For each case the students spent approximately 25–30 min working through each scenario, followed by a discussion led by a nursing instructor. Cases: appropriate dosage form modification for a patient who is unable to swallow. The other two cases focused on the compatibilities of IV medication administration.	The learning design incorporated facilitator interaction to help students reflect on the simulation experience. In these scenarios, the simulation used a 'pause and discuss' interaction between the facilitator and students as well as a structured debrief at the end of the session.	themes were identified from the analysis of the pharmacy student reflections: (1) apprehension regarding their preparedness to contribute to the exercise and recognition of their need to be more prepared for such situations, (2) enjoyment in participation, (3) their understanding of the value of collaboration between the two groups of students. Pharmacy students indicated that they felt prepared for the experience in the survey and yet reflected recognition of the need for additional preparation as well Explaination of this could relate to cognitive load and consequent anxiety may have been reduced with the review of salient features of the case scenarios and an understanding of the process and expectations for each scenario in advance of the day of simulation. Another possible explanation is that pharmacy students were asked to provide advice and guidance around medication administration to	Both nursing and pharmacy students appreciated the opportunity to work with students from another health discipline and recognized the importance of interprofessional collaboration to improve patient care. The desire for more interprofessional education activities expressed by both groups of students		not discussed	not discussed	no discussion re case design. Co-facilitated sessions	
73	Lockeman	The effect of an interprofessional simulation-based education program on perceptions and stereotypes of nursing and medical students: A quasi-experimental study.	Nurse education today	2017	USA	aim of our study was to explore whether a series of IPSE experiences promoted changes in attitudes and stereotypes among nursing and medical students.	Experimental study	Medicine + Nursing	Full body manikin	Their goal was to design scenarios that routinely occur in practice and require extensive team communication. In contrast to preceding studies, however, this learning experience was focused on the care of critically ill patients rather than nurse-physician communication.	grouped into interprofessional teams for a two-week period and participated in three two-hour simulations focused on collaboration around acutely ill patients. At the beginning of the first session, they completed a pretest survey with demographic items and measures of their perceptions of interprofessional clinical education, stereotypes about doctors, and stereotypes about nurses. They completed a posttest with the same measures after the third session. students were briefed by the faculty about Advanced Cardiac Life Support (ACLS) algorithms, and they worked as a team through six simulated resuscitation events using high-fidelity mannequins. Team communication was emphasized and profession-specific responsibilities were minimized. second and third sessions, teams worked to assess and treat simulated patients with an acute change in condition. Students were required to communicate effectively, think	After each scenario, the students were debriefed as a team by the faculty facilitator. Debriefing addressed the clinical aspects of the team's performance, with specific attention to whether appropriate interventions and escalation occurred when needed. Additionally, and perhaps more importantly, faculty facilitated a discussion about the teamwork aspect of the group's performance, with a focus on whether the team communicated effectively and worked well together to care for the deteriorating patient. The debrief was conducted using the behavioral checklist questions included in the		medical students showed little change from pretest to posttest on stereotypes of doctors, while nursing students had a significant increase in positive perceptions about doctors. No differences were noted between disciplines on changes in stereotypes of nurses. Our findings may demonstrate that IPSE has a differential impact on stereotypes that underlie interprofessional collaboration and define an individual student's interprofessional identity, or they may signify a need for interprofessional education that focuses more directly on the roles and responsibilities of each profession in a given setting in order to dispel negative stereotypes. Understanding what stereotypes evolve from traditional education and how interprofessional education can identify these perceptions		Since IPSE is resource-intensive, identifying the optimal dose, timing, and frequency of IPSE to support long-term change in practice is important.		collaborative. all three sessions, the faculty facilitators collaborated to create an instructor guide with details about each scenario and the expected course of interventions in order to promote consistency	
74	MacDonald	Emergency Management of Anaphylaxis: A High Fidelity Interprofessional Simulation Scenario to Foster Teamwork Among Senior Nursing, Medicine, and Pharmacy Undergraduate Students.	Cureus	2018	USA	HF-IPE scenario discussed in this report is designed to create a safe learning environment for undergraduate students to learn with and from one another while managing the care of an adult patient experiencing acute anaphylaxis	Descriptive study	Medicine + Nursing + Pharmacy		learner outcomes including enhanced knowledge of the roles of nursing, medical, and pharmaceutical professionals on the team, improved attitudes towards teamwork, and the demonstration of teamwork behaviors while caring for a patient experiencing anaphylaxis.	High fidelity simulation- case: managing the care of an adult patient experiencing acute anaphylaxis. mplementation of the simulation is divided into five phases: (1) baseline assessment, (2) early hypotensive reaction, (3) anaphylaxis, (4) recovery, and (5) resolution. The learning experience is student-centered, while the faculty play the role of facilitator and observer.					professional nursing, medicine, and pharmacy courses have rigid and busy schedules with little or no time for interprofessional activities, unless they can be scheduled within a course			case report of a simulation case design. No simulation completed and no data collected.
75	MacLeod	Measuring the effect of simulation experience on perceived self-efficacy for interprofessional collaboration among undergraduate nursing and social work students	Journal of interprofessional care	2022	USA	aim of this study was to examine the perceived self-efficacy of nursing and social work undergraduate students for interprofessional collaboration. The study question was: Does perceived self-efficacy of undergraduate nursing and social work students for interprofessional collaboration improve after a simulation-based interprofessional experience?	Mixed methodology	Nursing + Social work	Mixture	nursing students were expected to recognize appropriate clinical interventions for the patient and simultaneously collaborate with social work students to provide crisis intervention. Social work students were expected to utilize their empathic communication, rapport-building, assessment, crisis response and de-escalation skills to support the grandmother while collaborating with the nursing students.	simulation was originally designed as part of the critical care nursing curriculum and accordingly, students were presented with a trauma scenario involving an unconscious adolescent male admitted to the emergency department with a penetrating chest injury. Had to manage a paid actor who acted as a distressed family member.	The simulation was videotaped and viewed by faculty and students before a debriefing session. After the debriefing, students shared their perceptions about the experience	students valued the realistic nature of the simulation-based experience but also recognized that they were challenged by the high level of required competence. Students recognized that the simulation was stretching them beyond their competency level, yet they also acknowledged the importance of this for their learning.	hematic content analysis elicited three themes: (a) an improved understanding of the importance of communication and the interconnection of interprofessional roles; (b) a realistic simulation which challenged the students and (c) an increased appreciation for teamwork.	Our results may indicate that the success of the learning experience was more influenced by the positive patient outcome and the ability to review and reflect on the videotaped de-escalation of the family member than feelings of vulnerability and stress that the students experienced. The social work students' potential lack of comfort with the medical aspects of the	Care must be taken however to ensure adequate preparation of all participants and the provision of a psychologically safe environment that permits candid dialogue and support between participants. Caution when determining an environment for IPE simulation as some students e.g. social work have no medical background or clinical experience- can lead to feeling overwhlemened and affect psychological safety aspect		initially designed for critical care nursing course. Incorporation of the interprofessional component was a later addition to the simulation as the two programs began exploring interprofessional education opportunities.	

76	Mahmood	Interprofessional simulation education to enhance teamwork and communication skills among medical and nursing undergraduates using the TeamSTEPPS framework	Medical Journal Armed Forces India	2021	India	aims to evaluate an interprofessional simulation education (IPSE) module for undergraduate medical and nursing students on teamwork and communication skills using the TeamSTEPPS® framework. Simulation training using the Team Strategies and Tools to Enhance Performance and Patient Safety (TeamSTEPPS)	Mixed methodology	Medicine + Nursing	Other	The module was intended to improve interprofessional teamwork and communication skills among the participating team members	conducted at a simulation and skill centre in India. Details of natiure of simulation model used not detailed in article. Trauma simulation scenario. 5 phase module 1) pre simulation assessment checklist and immersed in simulation scenario. 2)participants engaged in team-building exercises and a didactic session and completed the interprofessional education collaborative (IPEC) competency self-assessment tool. 3)again immersed in simulated trauma scenarios and assessed for performance using the TeamSTEPPS® 2.0 Performance Observation Tool. After debriefing, students completed the post-training IPSE assessment checklist5 and the IPEC competency self-assessment tool. 4)collation of the qualitative data, which included retrieving audio recordings of the debriefing sessions in which open-ended questions were posed, and reviewing written reflections of the students with regard to the entire training experience. 5)two weeks later,	audio recordings of the debriefing sessions in which open-ended questions were posed		themes: need for IPSE in the curriculum, impact of structured tools for communication on patient safety, and awareness of the roles and responsibilities in interprofessional teamwork. Results: strongly suggestive of an improved awareness and attitude towards the interprofessional simulation session. strongly suggestive of improvement in teamwork and communication skills. participants strongly agreed that the simulation scenario helped them learn interprofessional team communication skills. A survey two weeks after completion of the module revealed that, to a great extent, participants held positive feelings toward interprofessional teamwork.		major challenge faced that was scheduling the sessions amidst the already-packed academic activities of both professions.	initial ice-breaker activities during the IPE didactic sessions and the trauma case scenarios were successful in engaging the participants, who had not previously attended any interprofessional sessions. Use of cases that are readily applicable to all professions involved.	IPSE module was conceptualised and developed by an interprofessional faculty team of twelve members from various specialities of medicine and nursing. The IP team sought to develop trauma case scenarios during the first two sessions of this module because they are interesting, relevant, realistic, and readily applicable to practice by both professions.
77	McLelland	Interprofessional simulation of birth in a non-maternity setting for pre-professional students	Nurse education today	2017	Australia	aims of the study were to examine whether an interprofessional team-based simulated birth scen ario would improve undergraduate paramedic, nursing, and midwifery students' self-efficacy scores and clinical knowledge when managing birth in an unplanned location. The secondary aim was to assess students' satisfaction with the newly developed interprofessional simulation.	Experimental study	Medicine + Nursing + Paramedics	Simulated participant	To enhance teamwork, interprofessional practice is dependent upon the individual practitioners' contribution of their own knowledge to the group's knowledge, and developing understanding of each other's roles.	setting: simulated hospital emergency department. Case: Interprofessional teams of five students managed a simulated unplanned vaginal birth. pre-briefing 15 min, scenario 30–40 min, and the debriefing up to 1 h. same professional actor for each birth	All of the academics were involved in the pre-briefing and the debriefing. The SP also provided feedback in the debriefing. The debriefing took place immediately following the simulation in a dedicated debriefing room opposite the Simulation Laboratory and was facilitated by two academics. Each simulation debrief consisted of semi-structured questions. The debrief concluded with a discussion of how the simulation could be applied to the respective participant's areas of clinical practice and what could be done in future to improve students" practice.	students' self-efficacy and confidence in ability to achieve a successful birth outcome was significantly improved at 1 month and remained increased at 3 months. Clinical knowledge was significantly increased in only one of three student groups: nursing (however, had had the least prior exposure to the management of a mother and baby after birth). All students were highly satisfied with the interprofessional simulation experience simulation.	improvement in self-efficacy scores pre and post the simulation, in relation to the subscales Interprofessional Interaction and Interprofessional Team Evaluation and Feedback		significant barrier for faculty; thus a cost-benefit analysis becomes an important step when considering when and where to embed interprofessional simulation into curricula. timetable clashes delayed the study and the logistics of including medical students (situated on another campus) prevented their inclusion.		The scenario was developed by a multi-disciplinary team of seven academics who developed the scenario including role profiles for each discipline: paramedic, nurse and midwife based on standard practices.
78	Hur	Patient safety interprofessional education program using medical error scenarios for undergraduate nursing and medical students in Korea	Journal of interprofessional care	2023	Korea	purpose of the current study is to determine the effectiveness of this patient safety IPE program and evaluate the program design and students' subsequent satisfaction levels.	Survey	Medicine + Nursing	Mixture	The scenarios were configured to include communication with patients, family members and caregivers, and health professionals in a responsive and responsible manner (i.e., SBAR communication [Situation, Background, Assessment, and Recommendation]; interprofessional communication practice); to use knowledge of one's own role and the roles of other health professionals to assess and patients' needs (roles and responsibilities); to apply the principles of team dynamics to perform effectively in different team roles (teamwork); and to practice with professionals from other disciplines while maintaining mutual respect and shared values (values for interprofessional practice).	Use of role play and high fidelity simulation- no details of each simulation means used. four scenarios, two (medication error scenarios) and the remaining two scenarios applied HFS to simulate oral prescription and blood transfusion errors. Each simulation 65 minutes. Each scenario was preceeded by prerequisite learning (45 minutes); Icebreaker/ISBAR practice (20 minutes) for interprofessional communication. structured pre-briefing (procedure guide and equipment introduction) was developed to establish psychological safety. Program involved integration of several teaching styles in IPE not just simulation- IPE program followed a highly structured and systematic approach, using teaching and learning methods such as case studies, role-playing, and simulation experience in an environment similar to the actual situation.	structured debriefing was facilitated to encourage reflection and generate meaningful conversation.		statistically significant difference in the RIPLS subscale scores for teamwork/collaboration and positive professional identity between pre- and posttests. students' attitudes and perceptions regarding their readiness to learn with other health professionals improved after the program. statistically significant difference in negative professional identity between the pre- and posttests among the medical students. no statistically significant difference in roles and responsibilities between the pre- and posttests.	patient safety motivation, there was a statistically significant difference between pre- and posttest scores	Timing within cirriculum- "It is a busy time for students to prepare for their final exams and future employment. So, I think I participated with a little less focus and preparedness."	medical and nursing students had different prior knowledge levels of patient safety; medical students studied patient safety subjects as part of their regular curriculum, but nurses did not. when conducting the IPE program of two departments, it is necessary to consider the integration of the subjects, the pre-learning course, and the timing of the program.	Five faculty members (three from the nursing department, and two from the medical department) with PhD degrees and experience in IPE teaching and research, patient safety, and simulation participated in the development of the patient safety IPE program. contents of the program were chosen according to the WHO curriculum guide (2010).
79	Millstein	Advance Care Planning and Communication Skills Improve after an Interprofessional Team Simulation with Standardized Patients	Palliative Medicine Reports	2022	USA	aims to evaluate an interprofessional approach to ACP (advanced care planning) education using SP (stimulated patient) encounters.	Mixed methodology	Medicine + Nursing + Social worker	Simulated participant	The first encounter demonstrated a patient interested in discussing ACP and open to completing ADs. The second encounter offered increased challenge, as the patient was focused on finding cures for a chronic illness rather than discussing ACP. The second encounter sought to enhance skills by having students negotiate those barriers.	three training modules and two SP encounters focused on ACP. training modules were delivered by the interprofessional faculty team, with curricular input from a health care lawyer and attendance by a certified hospital chaplain. Students were divided into interprofessional teams of two to four and participated in each of the two SP encounters consecutively, followed by team feedback sessions with each SP, and a large group debriefing run by the faculty.	large group debriefing run by the faculty.	Overall- significant improvements in ACP self-efficacy. Medium to large improvement in knowledge scores. Initially between baseline and after eduation module initial drop in self efficacy. Hypothesised- students initially overestimating their own ability to communicate about ACP issues effectively and subsequently recognizing the high complexity of ACP through the education modules and role play activities. This may be further explained by the fact that students were novice in their clinical experience, education, and exposure to ACP.	No change was detected for interprofessional teamwork	lack of improvement on students' ability to respond to patient emotions suggests that training modules and role-plays could be modified to better address patient affect.	SPs allows for less variability in student experience but may present a financial barrier		collaborative approach

80	Nystrom	Enacting simulation: A sociomaterial perspective on students' interprofessional collaboration	Journal of interprofessional care	2016	Sweden	aim of the study is to describe and analyse how interprofessional collaboration emerges in a simulated emergency situation as enacted by participating students.	Qualitative study	Medicine + Nursing	Full body manikin	aim of the simulation exercise was to provide opportunities for the students to practice professional skills as well as teamwork and collaboration	full day of simulation exercises. As part of the exercise, students were grouped into interprofessional teams that were kept together during the simulation day. medical and nursing students come together to practice in a simulated emergency situation, where a manikin is replacing the patient. All scenarios included were variations on the themes of acute emergency and/or deteriorating conditions of the patient. simulator was a human-sized manikin (site 1 used a Laerdal SimMAN 2 and site 2 used a Laerdal SimMAN 3G). Eighteen sessions of simulations were observed, and data were collected through standardised video recordings that were analysed collaboratively.	no debrief discussed	related to the manikin as a *technical body* , as they performed assessments in a way that the technical features of the manikin allowed. Second, they related to the manikin as a *physical/medical body* , as they performed their medical knowing in the situation, following the ATLS protocols. Finally, they related to the manikin as a *human body* , caring for the manikin like a real person, in their suspension of disbelief, for example, by tucking in a blanket around the feet in order to keep them warm.	student teams relate to the manikin as a technical, medical, and human body, and that interprofessional knowings and enactments emerge as a fluid movement between bodily positioning in synchrony and bodily positioning out of synchrony in relation to the sociomaterial arrangements. nterprofessional collaboration in the simulation room was enacted as bodily positionings in and out of synchronisation in a fluid way. When bodily positionings were in synchrony with the sociomaterial arrangements in interprofessional collaboration, the movements of the student team members were connected in a fluid chain of actions.The enactment of bodily positions in sync also showed that when the medical students performed their tasks, such as palpating the pulse and reporting the rate, the				no discussion surrounding scenario design	different focus to article interesting piece
81	O'Carroll	Interprofessional ArclightTM eye health workshop: impact on students' clinical identification and ophthalmic skills	Journal of interprofessional care	2023	Africa (Rwanda) (collaberation with university of St Andrews UK)	aim of this paper is to report on the impact of an interprofessional eye health workshop on healthcare students' clinical identification skills related to eye health, and self-reported confidence in ophthalmic skills.	Qualitative study	Medicine + Nursing + clinical medical officer + ophthalmic clinical officer	Task manikin	Basic eye anatomy, recognizing common eye diseases, and visual acuity assessment were common learning for all groups in these programs.	Arclight device, a low-cost ophthalmoscope and simulation eyes were used to enable students to practice ophthalmic skills and thereafter equip them. first part of the workshop consisted of students working in small interprofessional groups of four to five to: identify the similarities and differences in eye health teaching in each of their programs; explore their understanding of their respective professions' roles and responsibilities in relation to eye health; and to discuss what they perceived to be the enablers and barriers to ICP. The second part demonstrated how to use the Arclight device (AD), an inexpensive yet equally effective alternative to traditional direct ophthalmoscopes. Students examined and practised a range of skills on each other (Table 1) and used simulation tools that mimic all the major causes of blindness in the SSA setting.	no debrief discussed	students' ability to identify common eye conditions, and self-reported confidence in relation to all skills statistically improved post workshop, with some differences between professional groups in relation to eye health skills. Greatest increase seen in nursing students (least exposue to opthamology in course).	collaborative learning can influence positive perceptions of self-efficacy. ollaborative learning was particularly evident in the learning relationship between students and facilitators noted that OCO students were forthcoming in supporting their peers with learning new skills.		not discussed		content was carefully considered by an interprofessional faculty who brought a range of perspectives and awareness of students' prior knowledge and skills to ensure a quality and relevant IPE experience and to reflect the skills and knowledge reinforced by the Primary Eye Care training program in SSA	
82	Ogunyemi	Evolution of an obstetrics and gynecology interprofessional simulation-based education session for medical and nursing students	Medicine	2020	USA	purpose of this report is to describe the evolution and progression of an Obstetrics & Gynecology (OBGYN) IPE simulation program for medical and nursing students (NS) over a 4-year period. deductive investigational pathway that was initiated based on the hypothesis that a progressive IPE simulation program incorporating both faculty and interprofessional student collaboration would improve medical students' knowledge retention, comfort with procedural skills, positive	Survey	Medicine + Nursing	Mixture	major focus of this simulation session was the teaching of core competencies of Professionalism, Practice-Based Learning and Improvement, Interpersonal & Communication skills, and Interprofessional Collaboration	4 year development of cirrculum. Year 1: task model and whloe body model. students received a pre-curriculum lecture on intrapartum obstetrics and fetal heart rate tracings and watched a brief video on labor. The simulation was performed with students in groups of 3 to 4 rotating through three stations for 20 minutes each. At the station on simulated vaginal delivery, each student was guided in delivering a baby by MFM faculty with a simulation technician support using SimMom (Laerdal). An OBGYN resident gave an interactive workshop on fetal heart rate (FHR) tracings. Another OBGYN resident taught and assessed students on cervical dilation using "blinded" and "open" cervical models. In the subsequent 3 years ammendments made to the simulation set up and stations. An additional questionaire at 8 months post simulation added. we explored further possibilities of IPSE by creating a scenario in which medical students were able to learn	A debriefing session occurred at the end to answer questions and obtain constructive feedback.	Simulation improved students' short term medical knowledge, comfort, and perception with some long-term persistence at 4–8 months. Medical students' knowledge, comfort, and interest increased significantly post simulation. Outcome scores decreased but were still significantly improved at 4 months but nearly dissipated by 8 months. There were no significant differences between medical and NS self-assessment or faculty-assessment scores regarding IUD insertion, cervical examination, or contraception quiz scores.	ositive teamwork and respectful interaction occurred between the students.	we assessed relationships between simulation and interest in OBGYN by our perception survey. Results showed a very high interest immediately post-simulation but this decreased significantly at both 4 and 8 months. Many of the students' narrative comments stated that "they had forgotten" and "it was a long time ago". This finding suggests that interest in a			an interprofessional approach in curriculum development	
83	Oxelmark	Students' understanding of teamwork and professional roles after interprofessional simulation-a qualitative analysis	Advances in simulation	2017	Sweden	aims to understand if and how IPSE can change students' perception of each other's professions and their understanding of teamwork principles	Qualitative study	Medicine + Nursing	Full body manikin	Scenarios were designed for practicing teamwork principles and interprofessional communication skills by endorsing active participation by all team members. The explicit goals were to train (1) structured examination through ABCDE (Airway, Breathing, Circulation, Disability, Environment) and (2) structured communication through SBAR (Situation, Background, Assessment, Recommendation) and to give (3) feedback by applying closed loop communication and (4) attention to critical occurrences through "Speak up"	A lecture preceded the training to emphasize the principles of teamwork, structured communication, and systematic management of patients in everyday but potentially life-threatening situations. Five scenarios were designed. To promote active participation by the students from both disciplines, all scenarios included tasks that had to be performed by each profession, both separately and in collaboration. The scenarios comprised common medical conditions (for example, confusion after postoperative bleeding, vasovagal reactions, breathing problems, and anaphylactic reactions) in various healthcare settings that the students are likely to meet in their early professional career. The training sessions utilized a three-step model: briefing, scenario, and debriefing. Every student participated in three to five of the five scenarios during the day and observed the others. All students were introduced to the	The debriefing followed the model developed by Steinwachs [24]: first, the participants were asked to express their immediate feeling ("vent"). This was followed by a discussion that was divided into three steps: (1) what went well during the scenario, (2) what could be improved, and (3) what could be learned from this scenario. The initial focus on favorable actions was intended to establish a non-threatening situation and to serve as a basis for constructive discussion on what could be improved. Participants in the scenarios and observers were encouraged to engage in discussion and feedback.	students also expressed new insight into their own weaknesses and strengths in working with other professions, something that highlighted the need for continuous adaptation to each other in the team.IPSE increased their self-confidence.	The first question, aiming to identify changes in students' understanding of teamwork, resulted in three categories: (1)realizing and embracing teamwork fundamentals (very good detailed analysis of this-read full article ***) (2) reconsidering professional roles (The medical and nursing students expressed that they had a poor understanding of the others' professional roles before the simulation training, crucial experience was the importance of feeling free to cross each others' professional boundaries and to remind each other of important measures that otherwise might be forgotten (with potentially serious consequences for real patients) and that they were allowed to do so. nursing students voiced the importance for the medical students to feel supported by the rest of the team in the	described real emotional strain and feelings of stress and nervousness before the scenarios started. The stress was understood as positive for enabling learning and was described as different from the stress when taking an exam.	expectation that hierarchies and power relationships in healthcare are obstacles for achieving the learning goals of IPSE	A critical prerequisite for the development of these new insights was to feel confident in the learning environment. experienced the atmosphere during the simulation training to be kind, permissive, and non-judgmental. That all participants were students created a sense of equality and contributed to a positive and easy going ambiance. This made it easier to speak up, have the courage to express feelings of doubts, and be more outspoken. Important aspects that promoted a safe learning environment included working with a manikin, having clear learning goals given by the instructors, and encouraging the students to learn from their errors during simulation. It was also important that the team training consisted of several	collaborative design process	*Very relevant article *

84	Paige	Team Training of Inter-Professional Students (TTIPS) for improving teamwork.	BMJ simulation & technology enhanced learning	2017	USA	sought to determine whether SBT of interprofessional student teams (1) changes long-term teamwork attitudes and (2) is an effective form of team training.	Mixed methodology	Medicine + Nursing	Full body manikin	stressed the management of major trauma patients and nine team-based competencies (ie, shared mental model, role clarity, situational awareness, anticipatory response, resource management, open communication, cross monitoring, flattened hierarchy and mental rehearsal).	High-fidelity simulation-based training. two-scenario format with immediate after action debriefing. full-scale, computer-operated human patient simulator manikin (CAE, Montreal, Canada) was used. Equipment for trauma resuscitation was present. Sessions were recorded using METI Vision (CAE, Montreal, Canada).Three instructors typically led each session. Instructors also served as raters of team-based behaviours immediately after each scenario. Teams had three to eight students with at least two undergraduate nursing students and one medical student. Each team member assumed one of five main roles during the scenario: (1) primary nurse; (2) medication nurse; (3) chief resident; (4) airway physician and (5) intern physician. Scenario 1 involved resuscitation of a 30% total body surface area burn victim. Scenario 2 involved a blunt trauma victim with intra-abdominal haemorrhage and pneumothorax.	immediate structured debriefing	worthwhile nature centred around three themes: (1) clinical experience; (2) autonomy and (3) interprofessional collaboration. this experience revealed gaps in learning that could be addressed prior to the actual treatment of a trauma patient. autonomy in the SBT revealed the difference between passive care involvement of a patient versus actively 'calling the shots.'	Interprofessional education using high-fidelity simulation-based training of students is effective at teaching teamwork, changing interprofessional attitudes and improving long-term teamwork attitudes. expressed a satisfaction in working with members of another healthcare profession as part of their curricular programme in their respective school. They felt that it helped build bridges and clarify roles. demonstrated effectiveness on three of four Kirkpatrick Levels. Students had a very positive reaction to the SBT experience (level 1); they learnt team-based KSAs (level 2) and developed more positive attitudes towards each other's profession (level 2) and they incorporated improvements in attitudes related to team structure over the course of the clinical training period measured (level 3). this	Students over estimated self and team member performance when compared to observers scores. overestimation of self-performance by clinically inexperienced learners is an important finding, since it can help facilitators of debriefings calibrate remarks and evaluations to promote student learning by accurately reflecting	differing academic schedules of the Schools of Medicine and Nursing and the disproportionate sizes of the classes prevented all students from participating in the IPE SBT pilot.	not described	
85	Paige	Improvement in student-led debriefing analysis after simulation-based team training using a revised teamwork assessment tool.	Surgery	2021	USA	hypothesized that the use of the Q-TAS (quick team assessment scales) by students after SBT would improve the quality of their debriefings compared with the longer TAS.	Quantitative study	Medicine + Nursing	Full body manikin		dual-scenario simulation-based training session. SCENARIOW: The first was an urgent laparotomy for a stab victim with a lacerated iliac artery. The second was a case of lidocaine toxicity associated with a scalene regional block in a patient with an arm mass. Scenarios ran on a computerized mannequin (CAE, Montreal, QE). The students would undergo orientation to the simulated learning environment, including review of objectives and establishment of ground rules. They would then participate as an interprofessional OR team in a first SBT scenario. A facilitator-guided, structured debriefing immediately followed this first. After this debriefing, the interprofessional student teams would then perform a second scenario to practice what they had learned. Immediately after this second scenario, the interprofessional teams would then conduct a self-guided debriefing using either the TAS or the Q-TAS as a script.	immediate after-action debriefings after each scenario. In 2018, student teams conducted the second debriefing using as a guide the teamwork assessment scale, an 11-item, 3-subscale, 6-point Likert-type instrument. In 2019, they used a shortened, revised, 5-item version of the teamwork assessment scale, the quick teamwork assessment scale. A facilitator-guided, structured debriefing immediately followed this first scenario. It focused on key team-based competencies for effective team interaction.		One of the benefits of using the Q-TAS in dynamic clinical settings becomes apparent in this light. Its shortened format would provide clinicians enough time to complete it immediately after a clinical event, one of the best times to do a debriefing. Another benefit of the use of the Q-TAS in debriefing is the potential dual purpose it can serve. First, its use as a guide provides a best practice for clinical teams related to enhancing the effectiveness of their debriefings.16 Second, as a teamwork assessment tool, it can help clinical teams focus on improving interprofessional collaboration,	use of a shortened teamwork assessment instrument as a debriefing guide for student teams in student operating room team training was more effective in analysis of actions than the original, longer tool.	Imbalance of students due to logistically issues		Relevant piece but focus of the article in the debrief and use of a debrief tool
86	Peterson	Exploring Hidden Curricula in an Interprofessional Intensive Care Unit Simulation	CLINICAL SIMULATION IN NURSING	2018	USA	purpose of the study was to determine if students left a simulation having gained knowledge in areas other than our predetermined learning objectives.	Survey	Medicine + Nursing + physician assistant + respiratory therapy + clinical laboratory science + physical therapy + nuclear medicine technology	Full body manikin	investigate the hidden curriculum in an interprofessional (IP) intensive care unit (ICU) simulation. Predetermined learning objectives of the simulation were for students to recognize and manage care of the deteriorating ICU patient and appropriately respond and to demonstrate the use of teamwork and communication strategies in an IP health care setting.	setting: hospital-based simulation center that was previously an ICU. Each simulation session lasted two hours and was conducted six times to accommodate the number of students. A 15-minute prebrief was conducted at the beginning of the session. Each profession received shift reports as they entered the simulation. Six scenarios (Table) took place simultaneously, each with a different diagnosis. Scenarios ran for 45 minutes	concluded with a 15-minute in-room debriefing focusing on case-specific information. At the end of the in-room debriefing, all learners gathered for a large-group debriefing focusing on IP concepts. A discipline-specific debriefing was given after the large-group debriefing, allowing students to confer with faculty on any questions or concerns before leaving the center. All students participated in each of the three debriefings, which allowed for reflection about personal and professional contribution in the ICU setting.	three themes emerged which were not included in the learning objectives: (a) IP role clarity, (b) trust, and (c) self-efficacy. The subthemes for self-efficacy were confidence and anxiety or stress. Students revealed that they had varying confidence levels about applying knowledge or working in a team in a health care setting and questioned their abilities to handle anxiety and stress. Students across professions expressed that they learned the need for self-confidence. Some simply claimed that they learned the need for confidence in general. Students consistently stated that learning how to cope with the stressful nature of the health care environment and one's own feelings of anxiety and apprehension was a beneficial result of the simulation experience.	Many students who participated in this simulation indicated they were unsure about how other professionals' roles might complement their own. Although a common theme was to learn about other professions, students felt the need to help others understand the roles of their own professions. students were not simply trusting that others would do their jobs efficiently; they also realized the importance of trusting that everyone on the team would be patient-centric.		Faculty must identify inadvertent learning to adjust educational activities to enhance positive outcomes and combat or correct information acquired that is contradictory to intended outcomes. Hidden curricula can exist even within the context of simulation.	not described	
87	Murphy	Partnering to provide simulated learning to address Interprofessional Education Collaborative core competencies	Journal of interprofessional care	2015	USA	objective of this pilot was to explore how interprofessional simulations (IPS) affected nursing and social work students' readiness for interprofessional learning and their perception of interprofessional strengths and challenges.	Experimental study	Nusing + Midwifery	simulated participant	One simulation that addressed several competencies in the values and ethics domain	Four simulations were developed and implemented. Each simulation addressed several competencies in the four domains. Professional actors played the role of the family member who presented an ethical dilemma. Scenario 1- elderly patient with dementia presents with UTI. The other three simulations included: an asthmatic who had a same sex partner in the room asking many questions, a patient who coded with his distraught wife in the room interfering with care and a homeless man without a pulse (who does not recover) accompanied by his drug addicted friend.	In the debriefing SW and nursing faculty collaboratively facilitated a reflective discussion with all students focused on the IPEC core competencies and processed the realities of healthcare outcome as a team. All simulations were collaboratively debriefed in the same way immediately following the simulation.		student groups showed a significant change in attitudes toward interprofessional learning in that attitudes became significantly more positive after the simulations. Themes identified from the open-ended questions include communication, ability to work as a team, attitudes, listening skills and leadership skills. unique strength of interprofessional learning identified by nursing students was patient-centered care, while only SW students identified cultural competence, ability to work in a high-stress environment and compassion. On post-test both nursing and SW students identified new strengths such as reflective listening and open-mindedness for the SW role. The most frequently reported challenge by both groups on pre-test was interprofessional role uncertainty, while at post- [illegible]	it is the researchers' perspective that these simulations led students to be more reflective and acknowledge how little teamwork skills they possessed and were able to execute. After reflection, what they initially had identified as strengths were now reported as challenges.	participant being made volunatry vs complusory in different professions taking part	Nursing and social worker (SW) educators collaborated to revise existing simulations to incorporate SW and interprofessional components.	

88	Mutter	Feedback Methods in an Interprofessional Mock Paging Program	Medical science educator	2021	USA	This study addresses the feasibility of nursing student to medical student feedback, as well as the optimal method of feedback within an interprofessional mock paging program.	Qualitative study	M + N	simulated participant		Role play scenarios. Medical students were randomized to receive verbal feedback immediately after each of the three phone calls (intervention group) or delayed written feedback (control group) after the third phone call only. Specialty-specific case scenarios were developed and a single checklist for all scenarios was developed using the communication tool ISBAR. Medical students and nursing students had separate training sessions before the pages commenced. The nursing students administered the phone calls and evaluated the medical students by ISBAR checklist. An interrater reliability measure was obtained with physician observation of a selection of phone calls.	No formal debrief discussed by study design was group 1- immediate feedback from peers, group 2- delayed feedback from peers	mock paging programs can be used to help improve communication strategies and management for internship/clinician readiness	medical and nursing students voiced a desire to learn together more often. In this interprofessional context, an additional advantage of DP versus SRL is that students of different professions have the opportunity to offer each other constructive bidirectional feedback.	Immediate feedback significantly improves student checklist scores with a mock paging program. This finding suggests that coaching with feedback may have advantages above self-regulated learning.	logistically challenges - students were oriented to the activity in separate training sessions, due to schedule requirements		Three cases per specialty were developed and reviewed by faculty course directors	focus of the study on feedback- timing of feedback delivered in IPE
89	Musenge	Application of Interprofessional Education Model to University Pre-Licensure Health Students in the Management of Chronic Care Conditions in Zambia	Medical journal of Zambia	2022	Africa	we present the process of developing and piloting IPE modules on chronic conditions management for the undergraduate health professions training curriculum in Zambia.	Mixed methodology	Medicine + Nursing + Physiotherapy		The six competence domains include interprofessional communication, client-centered care, role clarification, team functioning, collaborative leadership, and interprofessional conflict resolution.	Modules for Hypertension, Diabetes Mellitus, Stroke, HIV, PTB, Cervical Cancer and Prostate Cancer were successfully developed and piloted among Medical, Nursing and Physiotherapy students. Case simulations were utilised to ensure the participation of the learners during the training. After module writing was completed, all the seven modules were printed in readiness for the pilot training. didactic program emphasizes interprofessional team building skills, knowledge of professions, patient centered care, service learning, the impact of culture on healthcare delivery and an interprofessional clinical component. The community-based experience demonstrates how IPCs provide service to patients and how the environment and availability of resources impact one's health status. The interprofessional-simulation experience describes clinical team skills training in both formative and summative		Our project demonstrates the utility of an IPE on student and highlights the potential importance of active interprofessional learning offerings. Some teaching and learning strategies used in other settings include seminars, workshops, small group discussions, role-playing exercises, clinical round discussions, journal clubs, simulations, team case conferences, clinical placement with clients. These strategies provide an opportunity to train healthcare students in a safe environment through observation, hands-on training, team interaction and critical feedback.24 It is clear that these strategies of IPE modify the attitudes of prospective healthcare professionals by exposing them to interactive communication, mutual respect and teamwork, thus facilitating the adoption of IPC in healthcare settings	The pilot revealed that students were able to identify the need to work together with other health professionals and they also acknowledged that this was key in provision of care to patients with chronic diseases. assisted students in understanding their own professional identity while gaining an understanding of other professional's roles on the health care team. During IPE, students focus on a collaborative approach to patient-centered care, with emphasis on team interaction, communication, service learning, evidence-based practice, and quality improvement	best fit model for implementing IPE for chronic care conditions at UNZA undergraduate program level appears to be the "didactic program, community-based experience, and interprofessional-simulation experience" models. Common assessment methods include knowledge, attitude, and practice questionnaires; clinical context tests such as	There are resistances to implementing interprofessional training programs among different professionals. Planning and coordinating the implementation of these programs may be challenging because of the differences in the culture of individuals and conflicts of interest. The lack of formal and academic experience in IPE and not being familiar with interprofessional training among the faculty. Financial constraints contributed to a reduction on the days for the pilot. Financial challenges are recognized barriers to the effective implementation of IPE programs. timing of the pilot was towards exam time for some of the students. This meant divided attention. Synchronizing calendars for the different disciplines of students is a recognized challenge to IPE. Other logistical challenges	students felt that there should be a form of reward system, most importantly certificates of attendance and to a lesser extent, an allowance of appreciation for contributing to the modules.	Multidisciplinary input at all levels of module creation culminated in content suitable for students from all the disciplines, while inherently warranting participation from the different professions for successful completion. The module developers themselves had to overcome several barriers to IPE to create modules that effectively promote IPC in patient care settings.	Not specifically simulation focused. Focus on creation of IPE teaching modules in which simulation may be a delievry strategy used. A lot of relevant content however on challneges of delivery IPE
90	Nystrom	Debriefing practices in interprofessional simulation with students: a sociomaterial perspective	BMC medical education	2016	Sweden	aim of the study is to explore how debriefing is carried out as a practice supporting students' interprofessional learning.	Mixed methodology	Medicine + Nursing	Full body manikin	aim of the simulation-based exercise was to provide opportunities for the students to engage in teamwork and interprofessional collaboration in a simulated clinical setting.	Eighteen debriefing sessions following interprofessional full-scale manikin-based simulation with nursing and medical students from two different universities were video-recorded and analysed collaboratively by a team of researchers, applying a structured scheme for constant comparative analysis. All scenarios included in the simulation were variations on the themes of acute emergency or deteriorating condition of the patient. The students at both sites had previously learned about acute emergency care. The simulations as well as the debriefings were recorded in their naturalistic setting	participated in the debriefing, either as active participants (two medical students and two to four nursing student per scenario) or as observers (four to six students per scenario) in the simulation scenario. The debriefing follows three steps, which is described by Steinwachs and can be found in many models of debriefing. The debriefing sessions generally lasted 15–30 min.		debriefing is intertwined with, and shaped by social and material relationships. Two patterns of enacting debriefing emerged. Debriefing as *algorithm* was enacted as a protocol-based, closed inquiry approach. Debriefing as laissez faire was enacted as a loosely structured collegial conversation with an open inquiry approach. neither an imposed structure of the debriefing, nor the lack of structure assured interprofessional collaboration to emerge. analysis shows how the emerging debriefing practices are relational to material objects, such as the protocols and arrangements of the debriefing room, but also to ideas of collegiality. if the aim is to support professional development and learning, it is important to promote a reflective practice in which the students get the opportunity to		The logistics act as a constraint on the debriefing, producing a need for the debriefing session to be completed within approximately 15 min. The time constraint is shown in the instructor's sayings, as repeated comments on the need to proceed or stop the debriefing. The time constraint might act negatively on, and jeopardise the potential for learning, both in the structured framing of debriefing as algorithm as well as in the loose structure and collegial discussion of debriefing as laissez-faire.			Focus on debrief. Good article on stratgies to approach IPE debrief in simulation. Limited IPE outputs in results
91	Nystrom	Observing of interprofessional collaboration in simulation: A socio-material approach	Journal of interprofessional care	2016	Sweden	study explores how social-material arrangements for observation of interprofessional collaboration in a simulated situation are enacted and how these observations are thematised and made relevant for learning.	Qualitative study	Medicine + Nursing	Full body manikin	aim of the simulation-based exercise was to provide opportunities for the students to engage in teamwork and interprofessional collaboration.	18 hours of video recordings and observational field notes of nursing and medical students engaged in simulations. All scenarios included in the simulation were variations on the themes of acute emergency or deteriorating condition of the patient. The simulations as well as the observations were recorded in their naturalistic setting. participated in the simulation session, either as active participants or as observers in the simulation scenario. SimMAN used. observing students were given specific tasks by the instructors. observations took place in two different kinds of sites as a part of the compulsory simulation session. In site 1, the students sat together with the instructor and the operator in a room where they could observe the simulation through a one-way screen. In site 2, the students sat in a separate room at a table, where they could watch the simulation live on a screen. These observation sites were two different socio-material arrangements.		1) Enacting proximal observation- the control room had a complex material set-up that positioned the observing students as a close up audience on a "stage within a stage". The metaphor refers to the students' presence in the same room as the instructor and the operator running the scenario. The observing students were participating in a stage where they had access and closeness to how the manikin was enacted by their fellow students. The observing students also became participants in the broader professional practice, in that they heard and witnessed how the instructor/operator answered the phone, and acted "as if" they were other professional actors in the hospital setting. In the observation room, the activities in the simulation room were often commented on in terms of "correct or incorrect" medical knowings, clinical issues, or professional behaviour. he students had the task to observe their fellow students,	findings show that the observation room was a location for normative professional judgements, more than for articulating interprofessional collaboration among the students. The students were participating in a passive, normative way as an audience and judges of what was or should be correct professional behaviour, and were "tested" of medical knowings and clinical issues. This conflicted with the task given to the observing students during the briefing, i.e. to observe communication, leadership, and good/less good professional performance of the team, as a focus of attention. These findings emphasise the need for educators of medical education and instructors of simulation-based education to reflect upon the briefing for the observing students.				Scenarios were not designed by researcher- preexisting the curriculum.	Focus on learning achieved as observer of stimulation

92	Paige	Moving Along: Team Training for Emergency Room Trauma Transfers (T2ERT2)	Journal of Surgical Education	2019	USA	To determine whether high fidelity simulation-based training (SBT) of interprofessional teams involving trauma transfers has an immediate impact on participants' team-based attitudes and behaviors.	Quantitative study	Medicine + Nursing	Full body manikin	we examined the impact of a high fidelity SBT curriculum targeting trauma team transfer from the resuscitation bay to the operating room.	2 hour dual scenario format. Each member of the team assumed predefined roles each of which had specific scenario respondsibilities. Roles were reversed on the second scenario among the undergraduate nursing students and the emergency medicine residents in order to allow them the opportunity to practice each role that they might undertake during trauma resuscitation. Scenario- blunt trauma victim.	immediate after action structured debriefing. debriefing focused on 9 team-based competencies (i.e., shared mental model (SMM), role clarity, situational awareness, anticipatory response, resource management, open communication, cross monitoring, flattened hierarchy, and mental rehearsal) as well as techniques for effective hand offs (i.e., Situation, Background, Assessment, Recommendation). At the conclusion of the session, each participant was asked to identify one team-based competency that he/she would attempt to practice/adopt in clinical practice.	what made the SBT worthwhile, comments centered around 3 main themes: (1) practice of clinical skills; (2) autonomy; and (3) interprofessional collaboration. Autonomy in the SBT helped them, as well as the emergency medicine residents, to have the chance to "call the shots" in a safe environment without consequences for patients.	Interprofessional trauma team transfer training using SBT changes attitudes toward key team-based competencies. articipants expressed a satisfaction in working with members of other disciplines and professions. For many nurses, this was the first experience in IPE, and they felt that it helped build bridges and clarify roles. For the surgery and emergency medicine residents, this SBT was an opportunity to work together and understand better each other's roles and responsibilities in trauma resuscitation. Within medicine: training allowed an opportunity for surgery residents and emergency medicine residents to work together outside of the actual clinical environment, helping them to understand better their roles and to work on providing effective feedback to one another.	Improving teamwork in the clinical setting requires breaking down long-held attitudes and beliefs that each profession has toward one another. These attitudes are deep-seated and are set early in professional development. To break down such barriers to effective teamwork, therefore, educators must intervene early in training to try to promote interprofession			standardized trauma resuscitation scenario was adapted from the existing third year medical student surgery clerkship SBT curriculum for the team training	
93	Peterson	Honors students in the health professions: An academic-practice partnership for developing interprofessional competencies through simulation.	Journal of professional nursing : official journal of the American Association of Colleges of Nursin	2021	USA	The primary aims of this academic-practice partnership project were to increase awareness of IPE, provide an experiential opportunity to learn the principles of interprofessional practice, and assess perceptions of readiness for practice. A secondary aim was to explore the motivations and learning expectations of undergraduate nursing and pre-medical honors students.	Survey	Medicine + Nursing	Simulated patient	Learning objectives for the simulation experiences and debriefing sessions were based on select sub-competency statements from four Interprofessional Education Collaborative (IPEC) core competency domains that are applicable across health care disciplines. Learning objective domains: Domain 1: Values/ethics for interprofessional practice. Domain 2: Roles/responsibilities (Less focus on this domain given the students' inexperience). Domain 3: Interprofessional communication. Domain 4: Teams and teamwork.	Students participated in four different simulations in 2 h, with 30 min allotted for the pre-brief, simulation, and debrief activities for each simulation. Four scenarios depicted discourse in health care such as incivility, workplace harassment, clinician impairment, and conflict with end of life care. A fifth scenario focused on disaster management and ethical decision-making with participants acting as victims of a school bus crash. The scenarios were intentionally designed to be interpersonally complex but didn't require discipline-specific knowledge related to clinical practice or patient care. Standardized patients fulfilled the roles of patients, family members, and other health care team members. In addition to a highly interactive learning environment, other best practices in honors education were employed including small group size, team-facilitation, opportunities for self-reflection, and a learner-centered focus within a [illegible]	Debriefings in the simulation room following each scenario provided opportunities for facilitator, peer, and self-assessments on individual and team performance and for learners to engage in a "take-two" to integrate feedback into the simulated health care scenario during a second replay of the same scenario. After both sub-groups completed the same scenario, faculty debriefed the experience in the simulation suite with all five participants and the standardized patient (s). A large group debrief lasting 15–20 min was conducted with all participants after scenario 5. A final large group debrief of all scenarios in a classroom [illegible]	The five predominant themes that emerged from the narrative data were: *Opportunity* , *Fun* , *Self-Awareness* , *Situational Awareness* , and *Value of Teamwork* . The theme of Opportunity contained sub-themes of Experiences and Learning. Participants attended to gain experience in working in health care teams; to increase exposure to health care in general and simulation-based learning; and to learn more about the academic-medical center that co-hosted the event. The theme of Self-Awareness included the sub-themes of Personal Responses, Seeing New Options for Situation, Speaking up Respectfully, Value of Practicing, and My Role. The theme of Situational Awareness included the categories of Awareness, Uncomfortable/Realistic Situations, and More Difficult to Do Than I Thought.	The theme *Value of Teamwork* included the categories of *Working Together* , *Value of Team* , and *Help My Teammate* . The concepts of collaboration and collaborative problem solving were prevalent in this theme. Sub themes of the Value of Teamwork that overlap with other themes include advocacy for self and others, increased awareness in the value of team, and awareness that conflict among team members is a real-world issue in healthcare. Learners also reported increased understanding of the role of others.	exposure to sensitive or uncomfortable topics such as incivility and ethical decision making increases awareness of "real-life" issues faced by multidisciplinary health care teams that pre-licensure students may not otherwise experience in the core curriculum or clinical experiences prior to entering the workforce. The opportunity to discuss sensitive topics in a simulated [illegible]	maintain an even number of nursing and pre-medical participants within each session, but relatively more premedical students	stimulating pedagogy, lecturer engagement, a safe learning space, shared experiences, and a low-stress environment are characteristics of enjoyable learning experiences. participation in the extracurricular activity on a weekend was voluntary, project leads were intentional in creating a comfortable, engaging, and fun environment; findings in this theme confirm this type of learning environment was present.	Nurse and physician educators from the university and the academic medical centre, including a Certified Health Simulation Educator, revised five simulation scenarios adopted from existing nursing, medical, and faculty development curricula.	
94	Popkess	Interprofessional Error Disclosure Simulation for Health Professional Students	CLINICAL SIMULATION IN NURSING	2017	USA	purpose of this study was to describe differences in knowledge and attitudes toward error disclosure after student participation in interprofessional simulation training.	Qualitative study	Nursing + Dentistry + Pharmacy	Simulated patient		Each interprofessional team participated in one disclosure simulation and observed two other teams where the standardized family member reacted in a relieved, angry, or sad/distrustful affect. The simulation experience required 2.25 hours of student time. event began with a 30-minute ice-breaker exercise designed to enhance team building and increase familiarity of team members. Eight university theater students were recruited and paid a nominal stipend to portray standardized family members during the simulation. Student teams prepared for the simulation experience by reviewing the case scenario and related background information in planning for the disclosure (30 minutes) and disclosing the error to the family member (five minutes for each team). Each team in the group performed an error disclosure with a standardized family member who was assigned one of the affects (relieved, angry, sad/distrustful). The [illegible]	Each of the first two simulations was followed by a ten-minute interprofessional, faculty-facilitated debriefing; the third simulation was followed by a 25-minute debriefing for allowing comparisons among the teams and the different standardized family member affects. At the conclusion, students spent 15 minutes completing the assessment instruments. An interprofessional team of two faculty served as debriefing facilitators using the framework	knowledge assessment indicated a significant improvement after completion of the simulation for all health disciplines. A significant improvement in most attitudes toward error disclosure was also demonstrated by each profession after the simulation. Students commented that the most valuable part of the experience was the ability to practice a real-life scenario and the time to reflect and debrief on the experience.	Students enjoyed working in teams with other health professions and appreciated learning the roles of the varied professions in their team		Ensurign scenarios applicable to all professions involved- Students commented that the scenario should be more applicable to dentistry. Debrief: feedback should be offered in a more positive way. No grade was tied to the preparation; therefore, some students may have placed different value on the need to complete the presimulation preparation. Dental students did not perceive the simulation or faculty feedback as positively as either nursing or pharmacy students, which may be due to a lack of perceived fidelity of the case scenario. Dental medicine is often practiced individually and does not involve routinely working with the professions represented in the simulation. In addition, the day of the week the simulation was offered may have been a factor in dental medicine students' perceptions. Dental medicine [illegible]	rained theater students as standardized family members provided fidelity and increased the authenticity of the simulation for students. simulation involved collaboration by trained, faculty in dental medicine, nursing, and pharmacy. Future offerings will include video recordings of the simulation to enhance student self-assessment skills and augment faculty facilitated debriefing feedback.		Quanittive study. No qualitative date to provide explainations behind findings
95	Seale	Combining medical, physiotherapy and nursing undergraduates in high-fidelity simulation: determining students' perceptions	BMJ Simulation and Technology Enhanced Learning	2019	UK	we sought to further explore the role of Hi-Fi SIM in IPE from the student's perspective by determining the attitudes of undergraduate students from each specialty towards simulation, IPE and human factors both before and after an IPE Hi-Fi SIM session.	Quantitative study	Medicine + Nursing	Full body manikin		10 min scenario consisted of the management of an acute clinical case focused on the skill set appropriate for a final year student from the relevant specialties. Remaining group members viewed the scenario via a live video link.	A group debrief facilitated by two clinically qualified tutors using the 'Debrief Diamond' structure9 followed each scenario	Phsyiotherapy students reported less previous exposure to simulation than medical or nursing students. physiotherapy students gave significantly lower scores for each of the five domains compared with nursing students and for the 'relevance of simulation' and 'communication' domains when compared with medical students. The significantly lower scoring by medical students on issues regarding situational awareness compared with nurses may be a consequence of a greater level of clinical exposure for nursing compared with medical students throughout their course, as suggested by the increased level of team-based work experience in the nursing group.						

96	Sigalet	Insight into team competence in medical, nursing and respiratory therapy students	Journal of interprofessional care	2015	Canada	primary objective was to understand more about the competence level of an undergraduate IP team with respect to leadership, roles and responsibilities, communication, situation awareness and resource utilization. Our second objective focused on examining the impact of adding a 30-min formalized TT module prior to engaging in a simulation-based TT curriculum on team performance.	Quantitative study	Respiratory therapy + Medicine + Nursing	Full body manikin	Team performance objectives focused on the concepts of leadership, roles and responsibilities, communication, situation awareness and resource utilization. Medical objectives focused on the basics of managing pediatric acute care to provide a context for team-training concepts. Students were expected to (i) assess the patient using the airway, breathing, circulation and disability assessment framework (ABCD), (ii) recognize life-threatening conditions such as tachycardia, hypotension and tachypnea with notable increased work of breathing, low oxygen saturations, altered level of consciousness and seizure activity, and (iii) provide effective medical management through consultation with their team inclusive of oxygen, fluids and	simulation-based TT curriculum consisted of two 20-min scenarios each followed by 40 min of structured debriefing. Four illness scenarios, sepsis and seizure for SimBaby™ (Laerdal Medical, Canada), and asthma and anaphylaxis for the METI™ (PediaSim HPS, Sarasota, FL) child manikin were scripted to provide one infant and one child scenario for each team. Scripts were purposely developed to engage all team roles; documenter, leader, procedure nurse, medication nurse and airway support. To mimic reality the medical student was asked to assume the leadership role, with nursing students assuming responsibility for medications, procedures, monitoring and documentation. The respiratory therapy student assumed responsibility for airway and breathing.	Debriefing followed a standard format including a reactions phase, an exploration phase where facilitator and participant observations and experiences were discussed as a group and a summary phase where students were encouraged to share what was meaningful and relevant to their learning	Individual analysis of item scores provides further insight into the relationship between specific teamwork concepts, performance and developmental readiness in ability to engage higher level team performance concepts providing information about the competence level of this level of learner, our first objective. Higher level skills may be easier to engage once lower level skills are mastered, allowing learners to direct psychological energy to more challenging team performance concepts.	study suggests a relationship between a simulation-based IP curriculum and improved team performance in teams of undergraduate medical, nursing and respiratory therapy students. suggest a relationship between blended learning using the pre-simulation video, discussion of team roles with subsequent simulation experiential learning and a significantly higher level of overall team performance. situational awareness, received the lowest score suggesting that the teams had little familiarity with this concept before engaging in the curriculum. this population of learners the use of simulation as a learning modality is optimized when it is supplemented with a didactic session on TT concepts. designing learning objectives educators should be cognizant of situation awareness being	From a theoretical perspective, we speculate that the formalized TT module delivered to these teams prior to engaging in the simulation-based TT curriculum boosted the team's cognitive knowledge about team training that resulted in significantly higher achievement scores	Students at this stage of educational development need to focus their energy, knowledge and skills on becoming more competent in their own role before they can expand their focus to support another team member role	Increasing the extent of curricula support and engaging more than one learning modality to deliver a curriculum should meet the needs of more learners. Thus, increasing foundational knowledge (cognitive entry behaviors) raises the potential for higher achievement	Scripts were purposely developed to engage all team roles; documenter, leader, procedure nurse, medication nurse and airway support.
97	Smith	Qualitative Analysis of Student Physical Therapist Reflective Writing: Does an Interprofessional Discharge Planning Simulation Increase their Understanding of the Role they play in Discharge Planning?	INTERNET JOURNAL OF ALLIED HEALTH SCIENCES AND PRACTICE	2020	USA	purpose of this study was to determine, by use of qualitative analysis of reflective writing, if an interprofessional discharge planning simulation will increase students' understanding of the role of physical therapy in the discharge planning process.	Qualitative study	Medicine + Nursing + Physio therapy + Soxial work	Simulated patient		students had a ten-minute pre-briefing meeting to discuss the case and complete a worksheet to help guide them in the meeting. facilitators were faculty members from nursing, social work, and physical therapy departments. The facilitators gave the instructions for the pre-briefing. Following the pre-briefing, the patient and his sister entered the room and the students had twenty minutes for the patient and family interaction. The facilitator(s) sat back silently during the patient simulated meeting. Each of the Sim-IPE DP sessions were recorded and placed on the e-learning platform for students to review and allow for self-reflection. DPT students were required to complete a reflection paper, using guiding questions to reflect upon.	Following the simulated meeting, the simulated participants left the room and each of the facilitators led a thirty minute debriefing.		Main themes 1) It is imperative to identify the main discharge issue particularly as it relates to patient safety and fall risk and to resolve this issue through the discharge planning process. (sub themes- Team communication is important when dealing with the discharge issue and Lack of team member preparedness negatively impacted the ability of the team to prioritized discharge issue). 2) Roles and responsibilities of an interprofessional discharge planning team (sub themes- Understanding of all providers' roles on the interprofessional team is essential, There is a need for PT providers to educate other members of the interprofessional team regarding their role.) 3) Gaps in knowledge with a lack of understanding of the discharge process.				The Sim-IPE discharge planning (DP) experience was designed and developed using the International Nursing Association for Clinical Simulation and Learning (INACSL) Standards of Best Practice: Simulation SM for Sim-IPE.
